# Osilodrostat improves blood pressure and glycemic control in patients with Cushing’s disease: a pooled analysis of LINC 3 and LINC 4 studies

**DOI:** 10.1007/s11102-024-01471-3

**Published:** 2025-01-25

**Authors:** Maria Fleseriu, Rosario Pivonello, John Newell-Price, Mônica R. Gadelha, Beverly M. K. Biller, Richard J. Auchus, Richard A. Feelders, Akira Shimatsu, Przemysław Witek, Marie Bex, Andrea Piacentini, Alberto M. Pedroncelli, André Lacroix

**Affiliations:** 1 Pituitary Center, Departments of Medicine and Neurological Surgery, Oregon Health and Science University, Portland, OR USA; 2 Dipartimento di Medicina Clinica e Chirurgia, Sezione di Endocrinologia, Università Federico II di Napoli, Naples, Italy; 3 School of Medicine and Population Health, University of Sheffield, Sheffield, UK; 4 Neuroendocrinology Research Center, Endocrinology Section, Medical School and Hospital Universitário Clementino Fraga Filho, Universidade Federal do Rio de Janeiro, Rio de Janeiro, Brazil; 5 Neuroendocrine and Pituitary Tumor Clinical Center, Massachusetts General Hospital, Boston, MA USA; 6 Department of Pharmacology, Division of Metabolism, Endocrinology and Diabetes, University of Michigan, Ann Arbor, MI USA; 7 Department of Internal Medicine, Endocrine Section, Erasmus Medical Center, Rotterdam, Netherlands; 8 Omi Medical Center, Kusatsu, Japan; 9 Department of Internal Medicine, Endocrinology and Diabetes, Medical University of Warsaw, Warsaw, Poland; 10 Department of Endocrinology, University Hospitals Leuven, Leuven, Belgium; 11 Recordati SpA, Milan, Italy; 12 Recordati AG, Basel, Switzerland; 13 Centre hospitalier de l’Université de Montréal, Montreal, Canada; 14 Present Address: Camurus AB, Lund, Sweden

**Keywords:** Cushing’s disease, Osilodrostat, Cortisol normalization, Hypertension, Diabetes, Long-term treatment

## Abstract

**Purpose:**

To evaluate the effect of osilodrostat and hypercortisolism control on blood pressure (BP) and glycemic control in patients with Cushing’s disease.

**Methods:**

Pooled analysis of two Phase III osilodrostat studies (LINC 3 and LINC 4), both comprising a 48-week core phase and an optional open-label extension. Changes from baseline in systolic and diastolic BP (SBP and DBP), fasting plasma glucose (FPG), and glycated hemoglobin (HbA_1c_) were evaluated during osilodrostat treatment in patients with/without hypertension or diabetes at baseline.

**Results:**

Of 210 patients, 82.9% met criteria for hypertension and 40.0% for diabetes at baseline. In patients with hypertension, reductions in mean SBP/DBP were observed from week (W)12 to W72, and 49.1%/58.5% of patients with high SBP/DBP (> 130/>90 mmHg) at baseline had normotensive levels at W72. Antihypertensive medication dose was reduced/stopped in 26.8% of patients, and the proportion taking antihypertensive medication decreased from 54.3% at baseline to 47.3% at W72. In patients with diabetes, mean FPG and HbA_1c_ decreased from W12 to W72, and 33.3%/61.5% with high FPG/HbA_1c_ (≥ 100 mg/dL/≥6.5%) at baseline had normal levels at W72. Antihyperglycemic medication dose was reduced/stopped in 35.7% of patients, and the proportion taking antihyperglycemic medication decreased from 21.9% at baseline to 17.1% at W72; improvements in SBP/DBP and FPG/HbA_1c_ were correlated with improvement in mean urinary free cortisol but not weight change. BP/glycemic parameters generally remained normal in patients without hypertension/diabetes at baseline.

**Conclusions:**

Patients with Cushing’s disease and comorbid hypertension/diabetes receiving osilodrostat had rapid and sustained improvements in SBP/DBP and glycemic control, respectively.

**Supplementary information:**

The online version contains supplementary material available at https://doi.org/10.1007/s11102-024-01471-3.

## Plain Language summary

**Why was this analysis carried out?:**

People with Cushing’s syndrome have higher-than-normal levels of the hormone cortisol. Cushing’s disease, the most common form of Cushing’s syndrome, is caused by a benign adenoma (non-cancerous tumor) of the pituitary gland. The adenoma produces too much of a hormone called adrenocorticotropic hormone (ACTH), which controls the production of cortisol.

Osilodrostat is a medicine that rapidly reduces and maintains normal cortisol levels in people with Cushing’s syndrome, including Cushing’s disease.

Many people with Cushing’s disease also have other medical conditions caused by high cortisol levels, such as high blood pressure and diabetes. We wanted to examine whether treatment with osilodrostat has any effect on blood pressure and blood sugar (glucose) levels in patients with Cushing’s disease.

**How was this analysis carried out?:**

The results of two previous studies of osilodrostat were combined to allow a larger number of people to be included over a long period.

Changes in blood pressure and glucose levels were measured during treatment with osilodrostat.

**What were the overall results?:**

In patients who had high blood pressure (hypertension) before starting osilodrostat, there was an overall reduction in blood pressure during osilodrostat treatment.

Some people who were taking medication for their hypertension were able to reduce the dose of their medication or stop it completely.

In patients who had normal blood pressure before starting osilodrostat, blood pressure remained normal during osilodrostat treatment.

In patients who had diabetes before starting osilodrostat, there was an overall reduction in glucose levels during osilodrostat treatment.

Some people who were taking medication for their diabetes were able to reduce the dose of their medication or stop it completely.

In patients with normal glucose levels before starting osilodrostat, glucose levels remained normal during osilodrostat treatment.

**What do the results mean?:**

By lowering cortisol levels, osilodrostat improves blood pressure in patients with hypertension and glucose levels in patients with diabetes; these effects were sustained over the long term.

This reduces the overall burden of disease for patients and may partly explain the improvement in quality of life reported in patients with Cushing’s disease treated with osilodrostat.

**Where can I find more information?:**

LINC 3 primary publication: Pivonello R *et al. Lancet Diabetes Endocrinol* 2020;8:748–61.

LINC 4 primary publication: Gadelha M *et al. J Clin Endocrinol Metab* 2022;107:e2882–95.

LINC 3 long-term publication: Fleseriu M *et al. Eur J Endocrinol* 2022;187:531−41.

LINC 4 long-term publication: Gadelha M *et al. Front Endocrinol (Lausanne)* 2023;14:1236465.

LINC 4 plain-language summary publication: Gadelha M. *Future Rare Dis* 2022;2:FRD28.

**Supplementary information:**

The online version contains supplementary material available at https://doi.org/10.1007/s11102-024-01471-3.

## Introduction

Cushing’s disease, the most common etiology of endogenous Cushing’s syndrome, is a rare disease characterized by hypercortisolemia resulting from an adrenocorticotropic hormone (ACTH)-producing adenoma [1–3]. Chronic exposure to excess cortisol has a substantial negative impact on morbidity, patients’ quality of life (QoL) and mortality [1, 4–10]. Furthermore, patients with Cushing’s disease have worse health-related QoL than patients with adrenal Cushing’s syndrome [11]. The main treatment goals are therefore to normalize cortisol levels, alleviate comorbidities and improve QoL [12, 13].

Hypertension and diabetes are common comorbidities of Cushing’s syndrome and are more prevalent in older (≥ 65 years) than younger (< 65 years) patients [14, 15]. At diagnosis, 80–85% of patients have hypertension [16], 31% have diabetes, and 19% have impaired glucose tolerance [14]. Furthermore, hypertension and diabetes persist in 64% and 44%, respectively, of patients with Cushing’s syndrome in remission [17].

Osilodrostat is a potent oral inhibitor of 11β-hydroxylase, which catalyzes the final step of cortisol synthesis [18]. In Phase III studies (LINC 3 [19] and LINC 4 [20]), osilodrostat provided rapid and sustained reductions in mean urinary free cortisol (mUFC), alongside improvements in signs, symptoms, and QoL that were maintained during long-term treatment [21].

We evaluated the impact of long-term osilodrostat treatment on comorbid hypertension and diabetes in a large, pooled population from the LINC 3 and LINC 4 studies.

## Methods

Full details have been published for LINC 3 (NCT02180217) [19] and LINC 4 (NCT02697734) [20]. Both were international Phase III trials, with 48-week core phases and optional extensions [19, 20].

### Patients

Adults aged 18–75 years with a confirmed diagnosis of persistent/recurrent (after pituitary surgery and/or irradiation) or *de novo* Cushing’s disease (non-surgical candidates), with mUFC > 1.5 times the upper limit of normal (ULN; LINC 3) or > 1.3 x ULN (LINC 4), were eligible for inclusion [19, 20].

The studies were conducted in accordance with the Declaration of Helsinki, and study protocols were approved by independent ethics committees/institutional review boards. Patients provided written informed consent to participate at the start of the core and extension phases.

### Study design

The 48-week core phase of LINC 3 included an 8-week randomized-withdrawal period for eligible patients (week [W]26–34) [19]. All patients received open-label osilodrostat treatment (starting at 2 mg twice daily [bid]), except for those randomized to placebo during the randomized-withdrawal period. Osilodrostat dose titration was permitted every 2 weeks until W12, then every 4 weeks.

The 48-week core phase of LINC 4 included an initial 12-week, double-blind, placebo-controlled period, during which patients were randomized to osilodrostat 2 mg bid or placebo [20]. Dose titration was permitted approximately every 3 weeks. At W12, patients restarted osilodrostat 2 mg bid (unless receiving a lower dose at W12). Patients on < 2 mg bid osilodrostat (or placebo) at W12 continued to receive the same dose, regardless of initial treatment allocation. Dose titration was permitted every 3 weeks thereafter.

In both studies, dose was titrated stepwise (2-5-10-20-30 mg bid), with decisions based on efficacy and tolerability.

Both studies permitted use of concomitant medications, including those for hypertension and diabetes.

### Assessments

#### Baseline hypertension and diabetes

For this *post hoc* pooled analysis, previously used definitions of hypertension and diabetes were applied [22, 23]. Patients were classified as having hypertension at baseline if they had: history of hypertension or antihypertensive medication use; systolic blood pressure (SBP) > 130 mmHg; and/or diastolic blood pressure (DBP) > 90 mmHg [22]. Patients were classified as having diabetes at baseline if they had: history of diabetes or antihyperglycemic medication use; glycated hemoglobin (HbA_1c_) ≥ 6.5%; and/or fasting plasma glucose (FPG) ≥ 126 mg/dL [23]. Glycemic status was defined as: FPG (mg/dL): normal, < 100; pre-diabetes (impaired glucose tolerance), 100–<126; diabetes, ≥ 126; HbA_1c_ (%): normal, < 5.7; pre-diabetes, 5.7–<6.5; diabetes; ≥6.5 [23].

#### mUFC

mUFC was calculated in central laboratories from the mean of two or three UFC samples (normal range 11–138 nmol/24 h [4–50 µg/24 h]) determined by liquid chromatography-tandem mass spectrometry.

#### Cardiovascular and metabolic-related parameters

Sitting SBP/DBP was assessed in LINC 3, supine SBP/DBP in LINC 4. Glycemic status was assessed by measuring FPG and HbA_1c_. Weight, body mass index (BMI), and waist circumference were assessed as risk factors for hypertension and diabetes.

### Safety assessments

Safety data have been published previously [19, 20, 24, 25].

### Statistical methods

Individual patient data from LINC 3 and LINC 4 were pooled and analyzed. Periods during which a patient was randomized to receive placebo were excluded. Changes in SBP, DBP, FPG, HbA_1c_, weight, waist circumference, and BMI were assessed at baseline, W12, W48, and W72 (common time points in both studies) according to presence/absence of hypertension/diabetes at baseline and presence/absence of antihypertensive/antihyperglycemic medication during the studies.

The following analyses were performed in populations with and without baseline hypertension and diabetes: baseline mUFC severity (x ULN; mild: mUFC < 2; moderate: mUFC 2–<5; severe: mUFC ≥ 5; these definitions are based on those used in a *post hoc* analysis of a Phase III study of pasireotide in patients with Cushing’s disease) [26] and mUFC control during the studies (controlled: ≤ULN; partially controlled: > ULN and ≥ 50% decrease from baseline; uncontrolled: > ULN with < 50% reduction from baseline). Changes in antihyperglycemic and antihypertensive medication were also assessed in patients with/without diabetes and hypertension at baseline.

Results were analyzed descriptively for all patients with an assessment at both baseline and the given visit. Pearson’s correlation coefficients were computed for data with normal distribution; r coefficients between 0.0 and 0.3 (or 0.0 and − 0.3), 0.3 and 0.7 (or − 0.3 and − 0.7), and 0.7 and 1.0 (or − 0.7 and − 1.0) represent weak, moderate, and strong positive (or negative) linear relationships, respectively. No other statistical testing was performed.

## Results

### Patient demographics and clinical characteristics

Overall, 210 patients (LINC 3, *n* = 137; LINC 4, *n* = 73) were included in the pooled analyses. At baseline, 82.9% (*n* = 174/210) of patients had hypertension and 40.0% (*n* = 84/210) had diabetes (Table 1). Mean baseline (standard deviation [SD]) mUFC levels (x ULN) were: 6.2 (10.0) in patients with hypertension and 4.4 (7.5) in those without; 6.4 (10.7) in patients with diabetes and 5.4 (8.9) in those without.

**Table 1:** Patient demographics and baseline characteristics

	Patients with hypertension *N* = 174	Patients without hypertension *N* = 36	Patients with diabetes *N* = 84	Patients without diabetes *N* = 126
Median age, years (min–max)	41.0 (19–70)	35.0 (19–55)	42.5 (19–69)	38.0 (19–70)
Sex, n (%)				
Female Male	134 (77.0) 40 (23.0)	33 (91.7) 3 (8.3)	69 (82.1) 15 (17.9)	98 (77.8) 28 (22.2)
Race, n (%)				
Caucasian Asian Black Native American Other Unknown	114 (65.5) 47 (27.0) 6 (3.4) 1 (0.6) 4 (2.3) 2 (1.1)	24 (66.7) 9 (25.0) 0 0 2 (5.6) 1 (2.8)	55 (65.5) 21 (25.0) 2 (2.4) 1 (1.2) 3 (3.6) 2 (2.4)	83 (65.9) 35 (27.8) 4 (3.2) 0 3 (2.4) 1 (0.8)
Weight, kg				
Mean (SD) Median (min–max)	80.6 (21.0) 75.0 (46.0–165.0)	76.3 (19.6) 71.6 (47.0–118.0)	83.8 (21.7) 75.0 (48.0–142.0)	77.3 (19.7) 74.3 (46.0–165.0)
Waist circumference, cm^a^				
Mean (SD) Median (min–max)	103.7 (17.4) 101.5 (67.0–158.0)	101.1 (22.3) 95.2 (67.0–171.0)	108.4 (17.6) 105.0 (78.0–171.0)	99.7 (18.1) 97.0 (67.0–158.0)
BMI, kg/m^2^				
Mean (SD) Median (min–max)	30.5 (7.3) 29.5 (18.0–56.0)	28.8 (7.2) 27.6 (19.0–51.0)	31.8 (7.8) 29.9 (20.0–56.0)	29.1 (6.7) 28.5 (18.0–51.0)
Median time to first osilodrostat dose since diagnosis, months (min–max)	52.5 (2–287)	65.9 (4–278)	38.8 (3–287)	64.1 (2–241)
Disease status, n (%)				
*De novo* Persistent/recurrent	19 (10.9) 155 (89.1)	1 (2.8) 35 (97.2)	10 (11.9) 74 (88.1)	10 (7.9) 116 (92.1)
Proportion of patients with previous pituitary surgery, n (%)	149 (85.6)	35 (97.2)	71 (84.5)	113 (89.7)
Proportion of patients with any previous medical treatment for Cushing’s disease, n (%)	148 (85.1)	28 (77.8)	71 (84.5)	105 (83.3)
Proportion of patients with previous pituitary irradiation, n (%)	26 (14.9)	5 (13.9)	13 (15.5)	18 (14.3)
Proportion of patients with comorbidities, n (%)	174 (100.0)	36 (100.0)	84 (100.0)	126 (100.0)
Mean number of comorbidities (SD)	10.8 (8.5)	6.5 (4.8)	12.7 (10.3)	8.3 (5.8)
mUFC				
Mean, nmol/24 h (SD) Mean, µg/24 h (SD)	848.8 (1384.1) 307.7 (501.7)	601.1 (1029.2) 217.9 (373.1)	888.7 (1475.1) 322.2 (534.7)	751.5 (1228.9) 272.4 (445.5)
Mean, x ULN (SD)	6.2 (10.0)	4.4 (7.5)	6.4 (10.7)	5.4 (8.9)
Median, nmol/24 h (min–max) Median, µg/24 h (min–max)	431.4 (21–9612) 156.4 (7.6–3484.4)	322.2 (67–5720) 116.8 (24.3–2073.5)	431.4 (21–9612) 156.4 (7.6–3484.4)	384.3 (47–9494) 139.3 (17.0–3441.6)
Median, x ULN (min–max)	3.1 (0.2–69.7)	2.3 (0.5–41.4)	3.1 (0.2–69.7)	2.8 (0.3–68.8)
Mean SBP, mmHg (SD)	135.3 (14.9)	113.2 (8.8)	133.1 (15.8)	130.6 (16.7)
Mean DBP, mmHg (SD)	86.5 (10.4)	74.6 (7.6)	83.6 (10.8)	85.1 (11.0)
FPG				
Mean, mg/dL (SD)^b^ Mean, mmol/L (SD)^b^	99.0 (26.8) 5.5 (1.5)	92.3 (22.1) 5.1 (1.2)	110.7 (32.3) 6.2 (1.8)	89.7 (17.1) 5.0 (1.0)
HbA_1c_				
Mean, % (SD) Mean, mmol/mol^c^	6.0 (0.9) 42.1	5.7 (0.8) 38.8	6.5 (1.1) 47.5	5.5 (0.5) 36.6

ULN for mUFC is 138 nmol/24 h (50 µg/24 h). ^a^*n*=170, *n* = 36, *n* = 83, *n* = 123, respectively; ^b^*n*=165, *n* = 35, *n* = 77, *n* = 123, respectively; ^c^SD not calculable

There were some differences between subgroups with and without hypertension or diabetes at baseline in the proportion of males, age, baseline mUFC, time to first dose since diagnosis, and number of comorbidities (Table 1). Baseline characteristics for patients with both, neither, or just one of hypertension and diabetes at baseline are summarized in Supplementary Table 1.

### Osilodrostat dose and exposure

In patients with and without hypertension at baseline, median (min–max) osilodrostat exposure was 101.2 (1–245) and 87.1 (6–204) weeks, respectively. Median (min–max) average osilodrostat dose was 6.2 (1–47) and 6.9 (1–21) mg/day, and median (min–max) dose given for the longest duration was 6.0 (0–60) and 6.0 (1–20) mg/day. Corresponding values in patients with and without baseline diabetes were, respectively: exposure, 102.6 (1–228) and 96.3 (1–245) weeks; dose, 6.1 (1–47) and 6.8 (1–46) mg/day; dose given for the longest duration, 6.0 (1–60) and 6.0 (0–60) mg/day.

### Change in blood pressure parameters over time

#### Change in SBP and DBP by presence/absence of hypertension at baseline

**Fig. 1:**
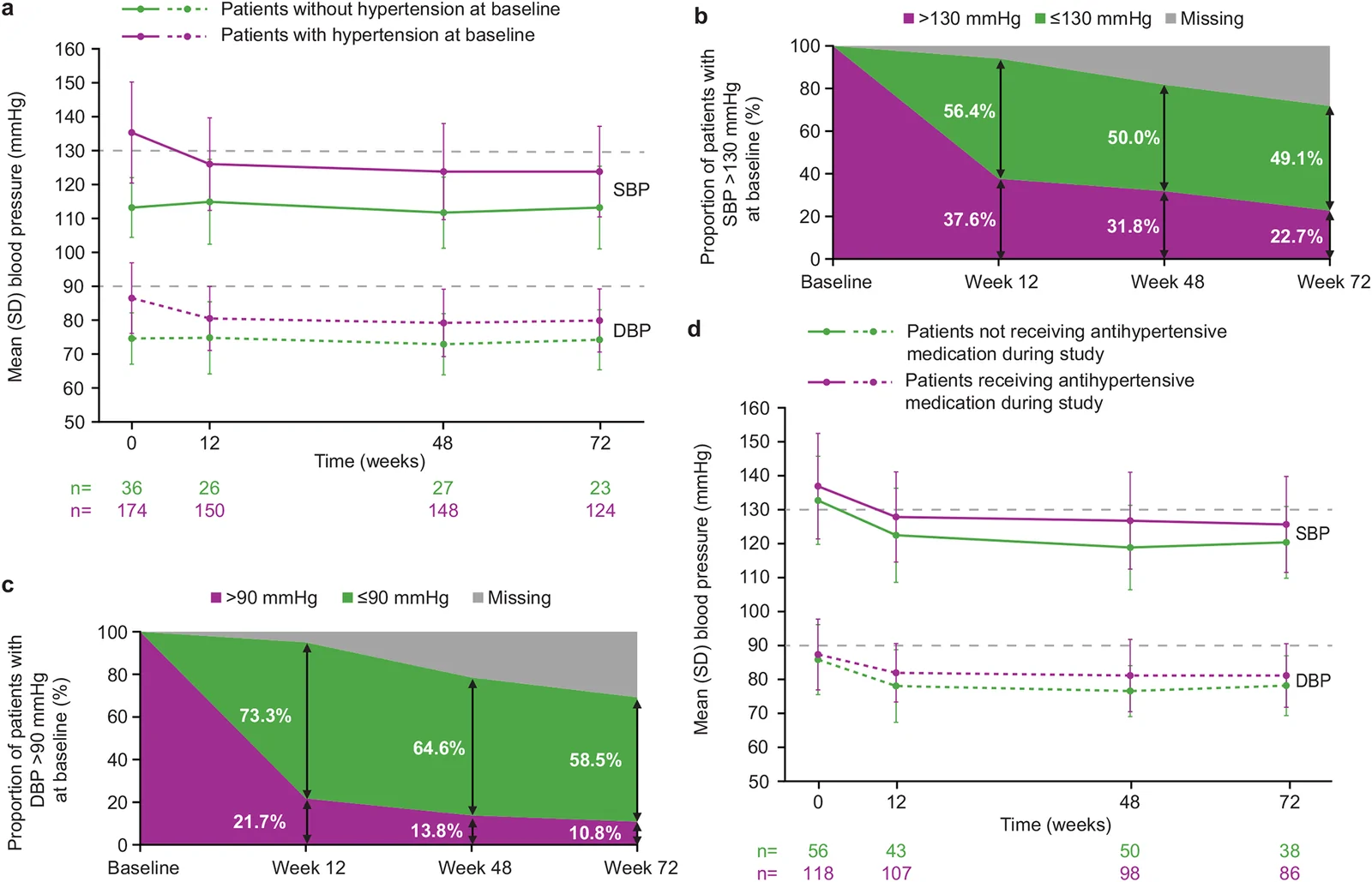
(**a**) Changes in mean SBP and DBP over time in patients with and without hypertension at baseline. Shift in (**b**) SBP and (**c**) DBP over time in patients with high blood pressure (SBP > 130 mmHg, DBP > 90 mmHg) at baseline. (**d**) Changes in mean SBP and DBP over time according to antihypertensive medication use during the studies in patients with hypertension at baseline. Dashed gray lines (panels a and d) represent upper limits beyond which hypertension is indicated: SBP 130 mmHg and DBP 90 mmHg

In patients with hypertension at baseline, SBP and DBP decreased from baseline (mean [SD]: 135.3 [14.9] and 86.5 [10.4] mmHg, respectively) to W12 (126.0 [13.7] and 80.5 [9.4] mmHg), and these reductions were maintained up to W72 (123.8 [13.4] and 79.9 [9.3] mmHg; Fig. 1a). In patients without hypertension at baseline, mean SBP and DBP remained normal during osilodrostat treatment (Fig. 1a).

In patients with SBP > 130 mmHg at baseline, 56.4% (*n* = 57/101) and 49.1% (*n* = 54/110) had SBP ≤ 130 mmHg at W12 and W72, respectively (Fig. 1b). Among those with DBP > 90 mmHg at baseline, 73.3% (*n* = 44/60) and 58.5% (*n* = 38/65) had DBP ≤ 90 mmHg at W12 and W72, respectively (Fig. 1c). Overall, 21.4% (*n* = 18/84) of patients with SBP ≤ 130 mmHg at baseline had SBP > 130 mmHg at W12, and 7.2% (*n* = 9/125) of patients with DBP ≤ 90 mmHg at baseline had DBP > 90 mmHg at W12. At W72, these proportions were 14.0% (*n* = 14/100) for SBP and 4.1% (*n* = 6/145) for DBP.

The largest decreases in SBP/DBP occurred in patients with the highest baseline SBP/DBP (see Supplementary Fig. 1 for further details).

Additional analyses assessed levels of 11-deoxycorticosterone, 11-deoxycortisol, and potassium in patients with versus without hypertension and/or hypokalemia at baseline (see Supplementary Figs. 2–4 for further details).

#### Change in SBP and DBP in patients with hypertension at baseline, by antihypertensive medication use

Overall, 54.3% (*n* = 114/210) of patients received antihypertensive medication at baseline, most commonly (≥ 10% of patients) amlodipine (14.9% [*n* = 17/114]), spironolactone (14.0% [*n* = 16/114]; all female), ramipril, and hydrochlorothiazide (both 13.2% [*n* = 15/114]). Of the 96 patients not taking antihypertensive medication at baseline, eight, 13, and 15 patients received antihypertensive medication at W12, W48, and W72, respectively. Twenty-three female patients started spironolactone during the study.

The proportion of patients taking antihypertensive medication declined over time (56.1% [*n* = 97/173] at W12, 47.3% [*n* = 61/129] at W72). At W12, 52.6% (*n* = 50/95) of patients had stopped/reduced their dose, 41.1% (*n* = 39/95) had no change, and 6.3% (*n* = 6/95) had increased their dose/started a new antihypertensive medication. Corresponding values at W72 were 26.8% (*n* = 19/71), 47.9% (*n* = 34/71), and 25.4% (*n* = 18/71), respectively. Of the 39 women treated with spironolactone (at baseline or during treatment), 32 stopped taking it during the studies.

In patients with hypertension at baseline, mean SBP/DBP decreased from baseline to W72, regardless of whether patients received antihypertensive medication during the studies (Fig. 1d). Reductions in mean SBP/DBP were similar irrespective of any change in antihypertensive medication over time (Supplementary Fig. 5).

#### Change in SBP and DBP by baseline mUFC severity and mUFC control

Baseline mean (SD) SBP/DBP in patients with hypertension at baseline was higher in those with severe mUFC elevation (> 5 x ULN; 138.1 [13.7]/89.5 [8.1] mmHg) than in those with mild (< 2 x ULN; 134.1 [15.6]/85.4 [9.4] mmHg) or moderate elevation (2–5 x ULN; 134.4 [15.2]/85.4 [11.8] mmHg) at baseline. Decreases in mean SBP/DBP over time were similar across all three baseline mUFC severity groups (Supplementary Fig. 6).

Of patients with hypertension at baseline, the proportions with mUFC ≤ ULN were 73.2% (*n* = 115/157) at W12 and 74.1% (*n* = 106/174) at W72. Corresponding proportions for patients without hypertension were 71.4% (*n* = 20/28) and 71.4% (*n* = 20/28), respectively. Patients with hypertension at baseline who achieved mUFC ≤ ULN or partial mUFC control at W72 had decreased SBP/DBP (Supplementary Fig. 7). Overall, there was a weak correlation between change in SBP/DBP and change in mUFC from baseline to W72 (*r* = 0.20, *P* = 0.048/*r* = 0.22, *P* = 0.028). There was no correlation between SBP/DBP and mUFC at W72.

Changes in antihypertensive medication in patients with hypertension at baseline, including a separate analysis for those receiving spironolactone, are shown in Supplementary Figs. 8 and 9.

#### Change in risk factors

Reductions in mean (SD) weight, waist circumference, and BMI were observed from baseline in patients with and without hypertension at baseline and were maintained over long-term treatment (see Supplementary Fig. 10 for further details).

### Change in glycemic parameters over time

#### Change in FPG and HbA_1c_ by presence/absence of diabetes at baseline

**Fig. 2:**
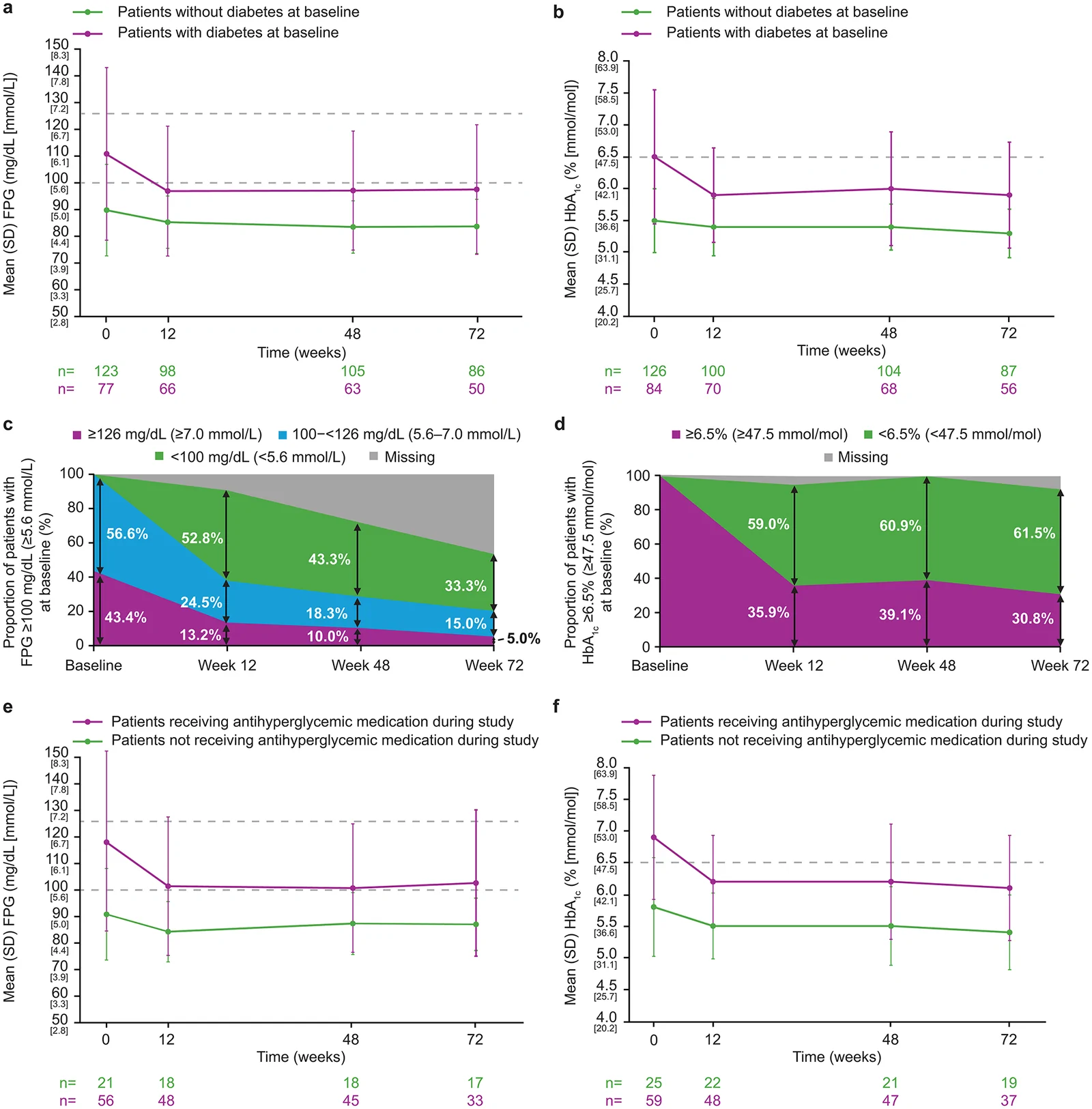
Mean (**a**) FPG and (**b**) HbA_1c_ over time in patients with and without diabetes at baseline. Shift over time in (**c**) FPG in patients with high FPG (≥ 100 mg/dL) at baseline and (**d**) HbA_1c_ in patients with high HbA_1c_ (≥ 6.5%) at baseline. Mean (**e**) FPG and (**f**) HbA_1c_ over time in patients with diabetes at baseline, according to antihyperglycemic medication use during the studies. For panels a and e, the dashed gray lines indicate the FPG thresholds for pre-diabetes (100 mg/dL [5.6 mmol/L]) and diabetes (126 mg/dL [7.0 mmol/L]). For panels b and f, the dashed gray lines indicate the HbA_1c_ threshold for diabetes (6.5% [47.5 mmol/mol])

In patients with and without diabetes at baseline, mean (SD) FPG decreased from 110.7 (32.3) and 89.7 (17.1) mg/dL, respectively, at baseline to 96.8 (24.3) and 85.2 (9.8) mg/dL at W12 and 97.4 (24.2) and 83.6 (10.1) mg/dL at W72 (Fig. 2a). Corresponding values for HbA_1c_ were 6.5% (1.1) and 5.5% (0.5), 5.9% (0.7) and 5.4% (0.5), and 5.9% (0.8) and 5.3% (0.4; Fig. 2b).

In patients with FPG ≥ 100 mg/dL at baseline, 33.3% (*n* = 20/60) had levels < 100 mg/dL at W72 (Fig. 2c). In those with HbA_1c_ ≥ 6.5% at baseline, 61.5% (*n* = 16/26) had levels < 6.5% at W72 (Fig. 2d). Overall, 5.7% (*n* = 7/123) of patients with FPG < 100 mg/dL at baseline had FPG ≥ 100 mg/dL at W12, and 0.7% (*n* = 1/137) of patients with HbA_1c_ < 6.5% at baseline had HbA_1c_ ≥ 6.5% at W12. At W72, these proportions were 4.3% (*n* = 6/140) for FPG and 1.7% (*n* = 2/120) for HbA_1c_.

The largest decreases in FPG and HbA_1c_ occurred in patients with the highest baseline values (see Supplementary Fig. 11 for further details).

#### Change in FPG and HbA_1c_ in patients with diabetes at baseline, by antihyperglycemic medication use

Overall, 21.9% (*n* = 46/210) of patients received antihyperglycemic medication at baseline, most commonly (≥ 10% of patients) insulin (73.9% [*n* = 34/46]), metformin (56.5% [*n* = 26/46]), metformin hydrochloride (41.3% [*n* = 19/46]), sitagliptin (13.0% [*n* = 6/46]), and liraglutide (10.9% [*n* = 5/46]). Among patients not taking antihyperglycemic medication at baseline, three and three patients received antihyperglycemic medication at W48 and W72, respectively.

The proportion of patients receiving antihyperglycemic medication generally remained stable from baseline to W12 (23.7% [*n* = 41/173]) then decreased over long-term treatment (W72: 17.1% [*n* = 22/129]). At W12, 48.0% (*n* = 24/50) of patients had stopped or reduced their dose, 50.0% (*n* = 25/50) had no change, and 2.0% (*n* = 1/50) had increased their dose or started a new antihyperglycemic medication. Corresponding values at W72 were 35.7% (*n* = 10/28), 53.6% (*n* = 15/28), and 10.7% (3/28), respectively.

In patients with diabetes at baseline, mean FPG and HbA_1c_ decreased from baseline to W72, regardless of whether patients received antihyperglycemic medication (Fig. 2e–f). Reductions in mean FPG and HbA_1c_ levels were similar in patients irrespective of any change in their antihyperglycemic medication (Supplementary Fig. 12).

#### Change in FPG and HbA_1c_ by baseline mUFC severity and mUFC control

**Fig. 3:**
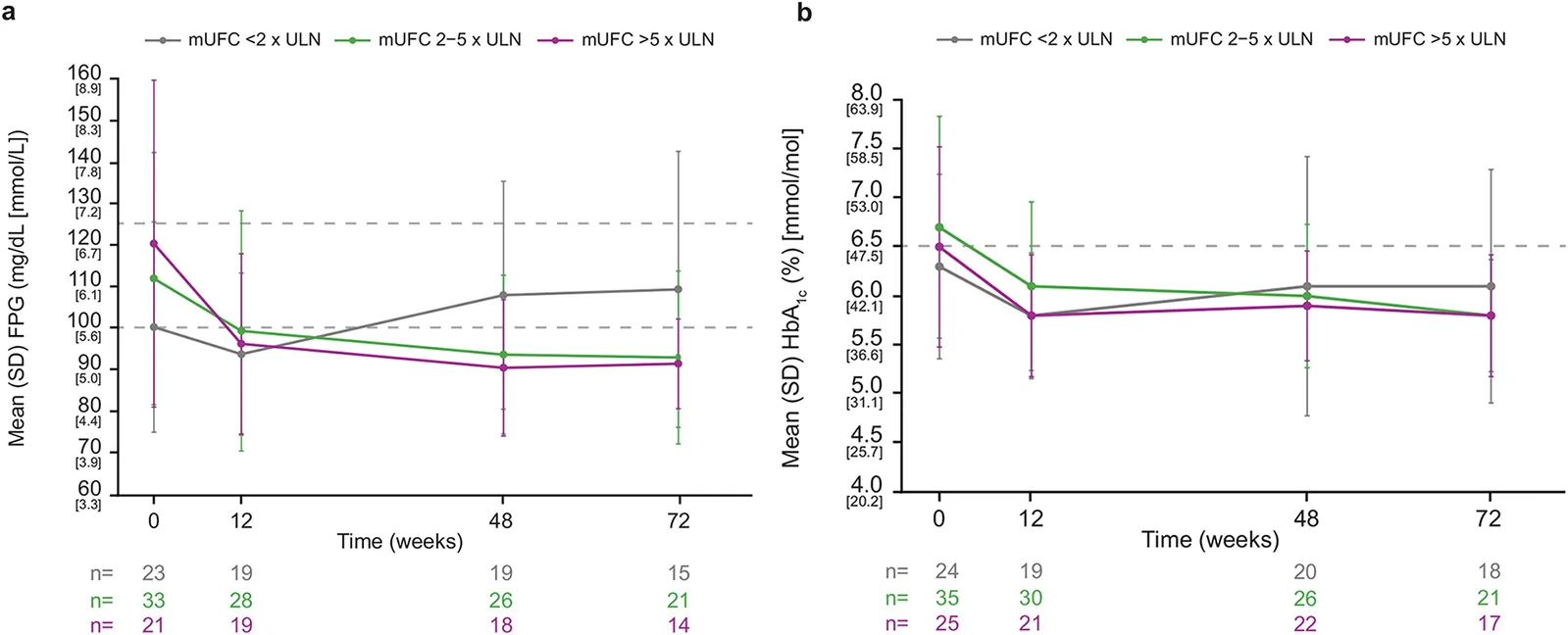
Mean (**a**) FPG and (**b**) HbA_1c_ over time in patients with diabetes at baseline, by baseline mUFC severity. For part a, the dashed gray lines indicate the FPG thresholds for pre-diabetes (100 mg/dL [5.6 mmol/L]) and diabetes (126 mg/dL [7.0 mmol/L]). For part b, the dashed gray line indicates the HbA_1c_ threshold for diabetes (6.5% [47.5 mmol/mol])

Baseline mean (SD) FPG and HbA_1c_ levels in patients with diabetes at baseline were higher in patients with severe mUFC elevation (120.3 [39.4] mg/dL and 6.5% [1.0], respectively) than in those with mild (100.2 [25.3] mg/dL and 6.3% [0.9]) or moderate elevation (111.9 [30.4] mg/dL and 6.7% [1.1]). Reductions in FPG were greater in those with baseline mUFC > 5 x ULN than in those with baseline mUFC 2–5 or < 2 x ULN. Reductions in HbA_1c_ were greater in those with baseline mUFC 2–5 and > 5 x ULN than in those with baseline mUFC < 2 x ULN (Fig. 3).

Of patients with diabetes at baseline, 76.6% (*n* = 59/77) and 71.0% (*n* = 49/84) had mUFC ≤ ULN at W12 and W72, respectively. Corresponding proportions for patients without diabetes were 70.4% (*n* = 76/108) and 75.5% (*n* = 77/126). In patients with diabetes at baseline, FPG decreased from baseline to W72 in patients with mUFC ≤ ULN but generally remained stable in patients with partial mUFC control or uncontrolled mUFC, while HbA_1c_ decreased from baseline to W72 irrespective of mUFC control (Supplementary Fig. 13). There was a weak correlation between changes in FPG and HbA_1c_ and change in mUFC from baseline to W72 (FPG: *r* = 0.31, *P* = 0.005; HbA_1c_: *r* = 0.21, *P* = 0.044). There was no correlation between FPG and HbA_1c_ and mUFC at W72.

In patients with diabetes at baseline who used antihyperglycemic medication during the studies, the proportion who reduced or stopped their dose was higher in those with mUFC ≤ ULN and partial mUFC control than in those with uncontrolled mUFC at W12 and W72 (Supplementary Fig. 14a). Changes in antihyperglycemic medication according to mUFC control in 12 patients without diabetes at baseline who started antihyperglycemic medication during the studies are shown in Supplementary Fig. 14b.

#### Change in risk factors

Reductions in mean weight, waist circumference, and BMI were observed from baseline in patients with and without diabetes at baseline, which were maintained over long-term treatment in both subgroups (see Supplementary Fig. 15 for further details).

## Discussion

This large, pooled analysis evaluated the effect of osilodrostat on long-term blood pressure and glycemic control in 210 patients with Cushing’s disease, 174/84 of whom had comorbid hypertension/diabetes at baseline. This is the largest prospective analysis of long-term changes in blood pressure, glycemia, and clinical parameters in patients with Cushing’s disease receiving long-term medical therapy.

Most patients (> 80%) had hypertension at baseline. In these patients, blood pressure improved during osilodrostat treatment; reductions in mean SBP/DBP were observed by W12 and were maintained over long-term (72 weeks) treatment. Notably, improvements in blood pressure were greater in those with higher baseline SBP/DBP. Overall, 49% and 58% of patients with high baseline SBP (> 130 mmHg) and DBP (> 90 mmHg), respectively, had normotensive levels (≤ 130 and ≤ 90 mmHg) at W72. Furthermore, blood pressure remained normotensive up to W72 in most patients without hypertension at baseline. Only a small proportion of patients who were normotensive at baseline had increases in SBP/DBP to > 130/>90 mmHg (14%/4%) at W72, one potential reason being accumulation of mineralocorticoid precursors [19].

Long-term hypercortisolism is associated with hypertension due to mineralocorticoid receptor activation, renin–angiotensin system activation, increased sensitivity to vasoconstrictors, increased sensitivity of beta-adrenergic receptors to catecholamines, suppression of vasodilators, sleep apnea, vascular rearrangement, and excessive fibrosis [27, 28]. Severe ACTH-dependent hypercortisolism can also significantly increase the levels of mineralocorticoids such as 11-deoxycorticosterone, which can lead to increased potassium excretion and more severe hypokalemia, thereby exacerbating the hypertensive effect [29]. In this study, mean baseline SBP/DBP was higher in patients with severe mUFC elevation (> 5 x ULN; comprising > 25% of the study population) than in those with mild (< 2 x ULN) or moderate mUFC elevation (2–5 x ULN). The proportion of patients with mUFC ≤ ULN during the studies was similar irrespective of the presence of baseline hypertension. Patients with mUFC ≤ ULN or partial mUFC control during the studies had greater reductions in SBP/DBP from baseline to W72 than those with uncontrolled mUFC. In addition, there was a weak correlation between change in mUFC and change in SBP/DBP from baseline to W72. These data demonstrate that rapid and sustained control of cortisol can improve comorbid hypertension during long-term osilodrostat treatment in patients with Cushing’s disease.

Over 50% of patients were taking antihypertensive medication at baseline. Blood pressure in patients with hypertension decreased between baseline and W72, irrespective of antihypertensive medication use. Some patients were able to reduce the dose of their antihypertensive medication or stop taking it completely. In patients taking spironolactone (all female), over 80% stopped taking it during the studies. Overall, more patients with mUFC ≤ ULN or partial mUFC control at W12 and W48 (but not at W72) were able to reduce the dose of their antihypertensive medication or stop taking it completely than those not achieving mUFC control. These results indicate that cortisol control leading to improvements in blood pressure allows reduction or withdrawal of antihypertensive medication [27]. Notably, despite the mechanism of action of osilodrostat and the increased mineralocorticoid precursors associated with treatment [30], relatively few cases of new/worsening hypertension or hypokalemia were reported during the studies [19, 20]. Nevertheless, blood pressure and potassium levels should be monitored periodically in patients receiving osilodrostat [31].

Overall, 40% of patients had diabetes at baseline. In these patients, glycemic control improved during osilodrostat treatment; reductions in mean FPG and HbA_1c_ were observed by W12 and were maintained over long-term (72 weeks) treatment. Improvements were greater in those with higher FPG and HbA_1c_ levels at baseline. In patients without diabetes at baseline, mean FPG and HbA_1c_ levels remained within normal levels up to W72. Overall, 33% and 62% of patients with high baseline FPG (≥ 100 mg/dL) and HbA_1c_ (≥ 6.5%), respectively, had normoglycemic levels (< 100 mg/dL and < 6.5%, respectively) at W72. Only 4% and 2% with normal FPG and HbA_1c_ at baseline, respectively, had increases to above normal at W72.

Hypercortisolemia is associated with impaired insulin action [32]. Levels of FPG and HbA_1c_ at baseline were greater in patients with severe mUFC elevation (> 5 x ULN) than in those with mild (< 2 x ULN) or moderate mUFC elevation (2–5 x ULN). Furthermore, those with greater mUFC elevations at baseline had greater reductions in FPG and HbA_1c_ during osilodrostat treatment. The proportion of patients with mUFC ≤ ULN during osilodrostat treatment was similar in those with and without baseline diabetes. Patients with mUFC ≤ ULN had greater reductions in FPG at W72 than patients with partial or uncontrolled mUFC. In contrast, HbA_1c_ decreased from baseline to W72 irrespective of mUFC control, although there was a weak correlation between change in mUFC between baseline and W72 and changes in both FPG and HbA_1c_. Although patients with diabetes at baseline had a higher mean weight than those without, the improvements in FPG and HbA_1c_ observed in patients with diabetes were not correlated with weight change. These data demonstrate that rapid and sustained control of cortisol can lead to improvements in comorbid diabetes in patients with Cushing’s disease during long-term osilodrostat treatment.

Over 20% of patients were taking antihyperglycemic medication at baseline. Glycemic control in patients with diabetes improved between baseline and W72 regardless of whether they received antihyperglycemic medication. Some patients were able to reduce the dose of their antihyperglycemic medication or stop taking it completely; this was more common in patients with mUFC ≤ ULN or partial mUFC control than in those not achieving mUFC control. These results indicate that cortisol control leading to improvements in FPG and HbA_1c_ allows reduction or withdrawal of antihyperglycemic medication [33].

Cardiovascular disease is one of the most common causes of death in patients with Cushing’s syndrome [7, 34, 35]. As obesity is a risk factor for cardiovascular disease, hypertension, and diabetes [36, 37], changes in weight-related parameters were evaluated in the current analysis. Mean weight, waist circumference, and BMI decreased in patients with and without hypertension and diabetes at baseline; this was sustained during long-term treatment. Weight loss during the study did not correlate with improvements in SBP/DBP or FPG/HbA_1c_, whereas improvements in cortisol did. This indicates that control of cortisol, not weight loss, was the driver of improved hypertension and diabetes; weight loss was a subsequent benefit. Despite complete biochemical remission, comorbidities such as hypertension, diabetes, and obesity persist in a subset of patients; this phenomenon is also observed in patients with Cushing’s disease after curative pituitary surgery.

Other studies of medical therapies have evaluated changes in blood pressure and glycemic parameters in patients with Cushing’s syndrome. These studies vary in terms of design, patient numbers, and treatment duration (see Table 2 for full details), and in most cases, outcomes are not reported according to the presence or absence of pre-treatment hypertension or diabetes. Levoketoconazole improved glycemic control but not blood pressure in those with Cushing’s syndrome [38, 39], while ketoconazole had no significant effect on either in patients with Cushing’s disease, with worsening hypertension reported in some cases [40]. Metyrapone was reported to improve blood pressure and glycemic control in patients with Cushing’s syndrome [41, 42], and subcutaneous pasireotide improved blood pressure in those with Cushing’s disease. Hyperglycemia is expected during pasireotide treatment [22, 43–45] based on its mechanism of action, but it is generally mild and reversible upon pasireotide discontinuation and can be effectively managed with antihyperglycemic medication and/or lifestyle changes [46–48].

**Table 2:** Studies reporting effects of medical therapies on blood pressure and glycemic parameters in patients with Cushing’s syndrome

Study	Study design and duration	Patient population	Treatment	Normalization of mUFC	Blood pressure outcomes	Glycemic outcomes
**Levoketoconazole**						
Fleseriu et al. (SONICS) [38]	Phase III, multicenter, open-label, single-arm study Titration period: 2–21 weeks Maintenance period: 6 months	94 patients with Cushing’s syndrome	Levoketoconazole 300–1200 mg/day	31–62% at the end of the 6-month maintenance phase, dependent on different evaluation criteria	71% had hypertension at baseline No significant change in SBP or DBP during study	38% had diabetes at baseline Baseline FPG: 103.6 mg/dL Baseline HbA_1c_: 6.0% Mean changes during study: –12.3 mg/dL and − 0.39% (both *P* < 0.0001) Of 16 patients with baseline HbA_1c_ ≥ 6.5%, 7 (44%) had levels < 6.5% at study end
Fleseriu et al. (SONICS extension) [52]	6-month extension to SONICS study	60 patients from SONICS titration period	Levoketoconazole 300–1200 mg/day	41% in patients with data at month 12	No significant change in blood pressure	Significant reduction (baseline to month 12) in FPG (–7.2 mg/dL, *P* = 0.02) but not HbA_1c_ (–0.3%, *P* = 0.068)
**Ketoconazole**						
Viecceli et al. [40]	Retrospective cohort study Duration of treatment: 2 weeks to 14.5 years	38 patients with Cushing’s disease	Ketoconazole 100–1200 mg/day	67% in patients with complete medical records	No significant change in blood pressure control Worsening of hypertension control in association with hypokalemia in some cases^a^	No significant change in glucose levels
**Metyrapone**						
Nieman et al. (PROMPT) [41]	Phase III/IV, multicenter, open-label, single-arm study Titration period: 12 weeks Extension period: 6 months	50 patients with Cushing’s syndrome	Metyrapone Final median dose: 1500 mg/day	47% at week 12	69% had hypertension at baseline Median SBP and DBP decreased by 4 and 5 mmHg, respectively, at week 12 Of patients taking antihypertensive medications at baseline, 10 (31%) had decrease and 5 (16%) had increase in number of antihypertensive medications during study	47% had diabetes at baseline Median FPG and HbA_1c_ decreased by 3% and 5% of baseline values, respectively, at week 12
Nieman et al. (PROMPT extension) [42]	6-month extension to PROMPT study	49 patients from PROMPT titration period	Dose not specified	49% at week 36 in evaluable patients	Clinical improvements continued^a^	Clinical improvements continued^a^
**Pasireotide**						
Colao et al. [43]	Randomized, double-blind, Phase III study 12 months	162 patients with Cushing’s disease	Pasireotide sc 600 µg or 900 µg twice daily	13% in the 600 µg arm and 25% in the 900 µg arm at month 12	Significant reduction in SBP (–6.1 mmHg) and DBP (–3.7 mmHg) from baseline to month 12 (both *P* = 0.03)	Blood glucose and HbA_1c_ increased soon after treatment initiation, then stabilized. Treatment with glucose-lowering medication initiated in 74/162 patients
Lacroix et al. [44]	Randomized, double-blind, Phase III study 12 months	150 patients with Cushing’s disease	Pasireotide im 10 mg or 30 mg every 4 weeks	35% in the 10 mg arm and 25% in the 30 mg arm at month 12	Mean change from baseline to month 12 in SBP was −4.6 mmHg in the 10 mg group and −5.0 mmHg in the 30 mg group DBP −3.4 and −3.1 mmHg	Mean FPG and HbA_1c_ increased within 1–2 months after starting pasireotide; ~50% of patients required initiation or adjustment of antihyperglycemic medication
Feelders et al. [45]	Phase II, open-label, multicenter study 35-week core phase Optional extension	68 patients with Cushing’s disease	Pasireotide sc (cabergoline added if cortisol remained elevated)	65% in patients on pasireotide monotherapy at week 35	In patients on pasireotide monotherapy: SBP/DBP at baseline: 125.7/82.7 mmHg (*n* = 26) SBP/DBP at week 99: 109.8/76.2 mmHg (*n* = 8)	FPG increased with pasireotide monotherapy during the first 8 weeks, then stabilized for remainder of study
**Mifepristone**						
Fleseriu et al. (SEISMIC) [53]	Multicenter, open-label study 24 weeks	50 patients with Cushing’s syndrome and diabetes/IGT or hypertension	Mifepristone 300–1200 mg/day	N/A	Patients with hypertension: Blood pressure response (decrease ≥ 5 mmHg) in 38% of patients (*P* < 0.05) No significant differences in mean SBP/DBP from baseline to week 24/end of treatment	In patients with diabetes/IGT: Glucose response (≥ 25% decrease in AUC_glucose_) in 60% of patients (*P* < 0.0001) Mean HbA_1c_ decreased from 7.4% to 6.3% (*P* < 0.001) FPG decreased from 149.0 to 104.7 mg/dL (*P* < 0.03)

^a^No further details given. AUC_glucose_, area under the glucose concentration–time curve; IGT, impaired glucose tolerance; im, intramuscular; sc, subcutaneous

Changes in glycemic parameters and blood pressure were assessed in an open-label study of mifepristone (SEISMIC) in 50 patients with Cushing’s syndrome and glucose intolerance/diabetes and/or hypertension. There were statistically significant reductions in HbA_1c_ and FPG in patients with impaired glucose tolerance/diabetes after 24 weeks. In patients with hypertension, 38% had a reduction in DBP from baseline of ≥ 5 mmHg [49].

Despite this analysis benefiting from the large, pooled population, allowing evaluation of an extended treatment period, there are limitations. Most patients enrolled in LINC 3 and LINC 4 had undergone previous pituitary surgery and/or received prior medical therapies, with longstanding hypercortisolism, resulting in differing potential to respond to osilodrostat treatment in terms of reversal of comorbidities. Furthermore, as patients with more severe hypertension or diabetes were excluded from the studies, we were unable to evaluate response to osilodrostat in such patients. Although the definition of hypertension (SBP > 130 mmHg and/or DBP > 90 mmHg) is consistent with that used for previous studies [22], it differs from the definition in published guidelines [50, 51]. As placebo-controlled periods were excluded from the pooled analysis, patients had different levels of osilodrostat exposure at various time points. In addition, patient numbers were small in some subgroups and at specific time points. Although both LINC 3 and LINC 4 permitted the use of concomitant medications for hypertension and diabetes, the effects of these relative to the cortisol-lowering effect of osilodrostat on lowering blood pressure and blood glucose parameters cannot be evaluated.

## Conclusions

Comorbid hypertension and diabetes improved in many patients with Cushing’s disease during long-term osilodrostat therapy, with many patients able to stop/reduce the dose of antihypertensive or antihyperglycemic medications. Sustained control of cortisol levels led to rapid improvements in blood pressure and glycemic parameters in patients with hypertension and diabetes at baseline. Notably, these improvements did not correlate with weight change. Comorbidities should be closely monitored as adjustments in concomitant medications are required for some patients taking osilodrostat when cortisol levels decline, including those who experience improvements in hypertension or diabetes. Overall, osilodrostat effectively controls hypercortisolism, leading to improved clinical signs, and may reduce treatment burden associated with comorbidities in patients with Cushing’s disease.

## Electronic Supplementary Material

Below is the link to the electronic supplementary material.

Supplementary Material 1

## Data Availability

The datasets generated and analyzed during the current study are not publicly available but are available from the corresponding author on reasonable request. Recordati Rare Diseases will share the complete de-identified patient dataset, study protocol, statistical analysis plan, and informed consent form upon request, effective immediately following publication, with no end date.

## References

[1] Lacroix A, Feelders RA, Stratakis CA, Nieman LK (2015) Cushing’s syndrome. Lancet 386(9996):913–927 10.1016/S0140-6736(14)61375-1 26004339

[2] Reincke M, Fleseriu M (2023) Cushing syndrome: a review. JAMA 330(2):170–181 10.1001/jama.2023.11305 37432427

[3] Gadelha M, Gatto F, Wildemberg LE, Fleseriu M (2023) Cushing’s syndrome. Lancet 402(10418):2237–2252 10.1016/S0140-6736(23)01961-X 37984386

[4] Braun LT, Vogel F, Reincke M (2022) Long-term morbidity and mortality in patients with Cushing’s syndrome. J Neuroendocrinol 34(8):e13113 10.1111/jne.13113 35312199

[5] Feelders RA, Pulgar SJ, Kempel A, Pereira AM (2012) The burden of Cushing’s disease: clinical and health-related quality of life aspects. Eur J Endocrinol 167(3):311–326 10.1530/EJE-11-1095 22728347

[6] Pivonello R, Isidori AM, De Martino MC, Newell-Price J, Biller BM, Colao A (2016) Complications of Cushing’s syndrome: state of the art. Lancet Diabetes Endocrinol 4(7):611–629 10.1016/S2213-8587(16)00086-3 27177728

[7] Mondin A, Ceccato F, Voltan G, Mazzeo P, Manara R, Denaro L et al (2023) Complications and mortality of Cushing’s disease: report on data collected over a 20-year period at a referral centre. Pituitary 26(5):551–560 10.1007/s11102-023-01343-2 PMC10539191 37495935

[8] Fleseriu M, Varlamov EV, Hinojosa-Amaya JM, Langlois F, Melmed S (2023) An individualized approach to the management of Cushing disease. Nat Rev Endocrinol 19(10):581–599 10.1038/s41574-023-00868-7 37537306

[9] Loughrey PB, Herron B, Cooke S, Weir P, Smyth JE, Mullan KR et al (2024) Insights on epidemiology, morbidity and mortality of Cushing’s disease in Northern Ireland. Endocr Relat Cancer 31(9):e240028 10.1530/ERC-24-0028 PMC11301418 38889004

[10] Rudman Y, Fleseriu M, Dery L, Masri-Iraqi H, Sasson L, Shochat T et al (2024) Endogenous Cushing’s syndrome and cancer risk. Eur J Endocrinol 191(2):223–231 10.1093/ejendo/lvae098 39067000

[11] Valassi E, Feelders R, Maiter D, Chanson P, Yaneva M, Reincke M et al (2018) Worse health-related quality of life at long-term follow-up in patients with Cushing’s disease than patients with cortisol producing adenoma. Data from the ERCUSYN. Clin Endocrinol (Oxf) 88(6):787–798 10.1111/cen.13600 29574994

[12] Fleseriu M, Auchus R, Bancos I, Ben-Shlomo A, Bertherat J, Biermasz NR et al (2021) Consensus on diagnosis and management of Cushing’s disease: a guideline update. Lancet Diabetes Endocrinol 9(12):847–875 10.1016/S2213-8587(21)00235-7 PMC8743006 34687601

[13] Nieman LK, Biller BM, Findling JW, Murad MH, Newell-Price J, Savage MO et al (2015) Treatment of Cushing’s syndrome: an Endocrine Society clinical practice guideline. J Clin Endocrinol Metab 100(8):2807–2831 10.1210/jc.2015-1818 PMC4525003 26222757

[14] Giordano C, Guarnotta V, Pivonello R, Amato MC, Simeoli C, Ciresi A et al (2014) Is diabetes in Cushing’s syndrome only a consequence of hypercortisolism? Eur J Endocrinol 170(2):311–319 10.1530/EJE-13-0754 24255133

[15] Amodru V, Ferriere A, Tabarin A, Castinetti F, Tsagarakis S, Toth M et al (2023) Cushing’s syndrome in the elderly: data from the European Registry on Cushing’s Syndrome. Eur J Endocrinol 188(4):395–406 10.1093/ejendo/lvad008 36749009

[16] Fallo F, Di Dalmazi G, Beuschlein F, Biermasz NR, Castinetti F, Elenkova A et al (2022) Diagnosis and management of hypertension in patients with Cushing’s syndrome: a position statement and consensus of the Working Group on Endocrine Hypertension of the European Society of Hypertension. J Hypertens 40(11):2085–2101 10.1097/HJH.0000000000003252 35950979

[17] Schernthaner-Reiter MH, Siess C, Gessl A, Scheuba C, Wolfsberger S, Riss P et al (2019) Factors predicting long-term comorbidities in patients with Cushing’s syndrome in remission. Endocrine 64(1):157–168 10.1007/s12020-018-1819-6 PMC6453862 30467627

[18] Bertagna X, Pivonello R, Fleseriu M, Zhang Y, Robinson P, Taylor A et al (2014) LCI699, a potent 11β-hydroxylase inhibitor, normalizes urinary cortisol in patients with Cushing’s disease: results from a multicenter, proof-of-concept study. J Clin Endocrinol Metab 99(4):1375–1383 10.1210/jc.2013-2117 24423285

[19] Pivonello R, Fleseriu M, Newell-Price J, Bertagna X, Findling J, Shimatsu A et al (2020) Efficacy and safety of osilodrostat in patients with Cushing’s disease (LINC 3): a multicentre Phase III study with a double-blind, randomised withdrawal phase. Lancet Diabetes Endocrinol 8(9):748–761 10.1016/S2213-8587(20)30240-0 32730798

[20] Gadelha M, Bex M, Feelders RA, Heaney AP, Auchus RJ, Gilis-Januszewska A et al (2022) Randomized trial of osilodrostat for the treatment of Cushing disease. J Clin Endocrinol Metab 107(7):e2882–e2895 10.1210/clinem/dgac178 PMC9202723 35325149

[21] Pivonello R, Fleseriu M, Newell-Price J, Shimatsu A, Feelders RA, Kadioglu P et al (2024) Improvement in clinical features of hypercortisolism during osilodrostat treatment: findings from the Phase III LINC 3 trial in Cushing’s disease. J Endocrinol Invest 47(10):2437–2448 10.1007/s40618-024-02359-6 PMC11392997 38696122

[22] Pivonello R, Petersenn S, Newell-Price J, Findling JW, Gu F, Maldonado M et al (2014) Pasireotide treatment significantly improves clinical signs and symptoms in patients with Cushing’s disease: results from a Phase III study. Clin Endocrinol (Oxf) 81(3):408–417 10.1111/cen.12431 24533697

[23] American Diabetes Association Professional Practice Committee (2023) 2. Diagnosis and classification of diabetes: standards of care in diabetes – 2024. Diabetes Care 47(Suppl 1):S20–S42 10.2337/dc24-S002 PMC10725812 38078589

[24] Fleseriu M, Newell -Price J, Pivonello R, Shimatsu A, Auchus RJ, Scaroni C et al (2022) Long-term outcomes of osilodrostat in Cushing’s disease: LINC 3 study extension. Eur J Endocrinol 187(4):531–541 10.1530/EJE-22-0317 PMC9513654 35980235

[25] Gadelha M, Snyder PJ, Witek P, Bex M, Belaya Z, Turcu AF et al (2023) Long-term efficacy and safety of osilodrostat in patients with Cushing’s disease: results from the LINC 4 study extension. Front Endocrinol (Lausanne) 14:1236465 10.3389/fendo.2023.1236465 PMC10482037 37680892

[26] Petersenn S, Newell-Price J, Findling JW, Gu F, Maldonado M, Sen K et al (2014) High variability in baseline urinary free cortisol values in patients with Cushing’s disease. Clin Endocrinol (Oxf) 80(2):261–269 10.1111/cen.12259 PMC4231220 23746264

[27] Barbot M, Ceccato F, Scaroni C (2019) The pathophysiology and treatment of hypertension in patients with Cushing’s syndrome. Front Endocrinol (Lausanne) 10:321 10.3389/fendo.2019.00321 PMC6536607 31164868

[28] Mancini T, Kola B, Mantero F, Boscaro M, Arnaldi G (2004) High cardiovascular risk in patients with Cushing’s syndrome according to 1999 WHO/ISH guidelines. Clin Endocrinol (Oxf) 61(6):768–777 10.1111/j.1365-2265.2004.02168.x 15579193

[29] Cicala MV, Mantero F (2010) Hypertension in Cushing’s syndrome: from pathogenesis to treatment. Neuroendocrinology 92(Suppl 1):44–49 10.1159/000314315 20829617

[30] Dougherty JA, Desai DS, Herrera JB (2021) Osilodrostat: a novel steroidogenesis inhibitor to treat Cushing’s disease. Ann Pharmacother 55(8):1050–1060 10.1177/1060028020968808 33143437

[31] Fleseriu M, Biller BMK (2022) Treatment of Cushing’s syndrome with osilodrostat: practical applications of recent studies with case examples. Pituitary 25(6):795–809 10.1007/s11102-022-01268-2 PMC9401199 36002784

[32] Kamba A, Daimon M, Murakami H, Otaka H, Matsuki K, Sato E et al (2016) Association between higher serum cortisol levels and decreased insulin secretion in a general population. PLoS One 11(11):e0166077 10.1371/journal.pone.0166077 PMC5115704 27861636

[33] Mehlich A, Bolanowski M, Mehlich D, Witek P (2023) Medical treatment of Cushing’s disease with concurrent diabetes mellitus. Front Endocrinol (Lausanne) 14:1174119 10.3389/fendo.2023.1174119 PMC10150952 37139336

[34] Limumpornpetch P, Morgan AW, Tiganescu A, Baxter PD, Nyawira Nyaga V, Pujades-Rodriguez M et al (2022) The effect of endogenous Cushing syndrome on all-cause and cause-specific mortality. J Clin Endocrinol Metab 107(8):2377–2388 10.1210/clinem/dgac265 PMC9282270 35486378

[35] Ragnarsson O, Olsson DS, Papakokkinou E, Chantzichristos D, Dahlqvist P, Segerstedt E et al (2019) Overall and disease-specific mortality in patients with Cushing disease: a Swedish nationwide study. J Clin Endocrinol Metab 104(6):2375–2384 10.1210/jc.2018-02524 30715394

[36] El Meouchy P, Wahoud M, Allam S, Chedid R, Karam W, Karam S (2022) Hypertension related to obesity: pathogenesis, characteristics and factors for control. Int J Mol Sci 23(20):12305 10.3390/ijms232012305 PMC9604511 36293177

[37] Hubert HB, Feinleib M, McNamara PM, Castelli WP (1983) Obesity as an independent risk factor for cardiovascular disease: a 26-year follow-up of participants in the Framingham Heart Study. Circulation 67(5):968–977 10.1161/01.cir.67.5.968 6219830

[38] Fleseriu M, Pivonello R, Elenkova A, Salvatori R, Auchus RJ, Feelders RA et al (2019) Efficacy and safety of levoketoconazole in the treatment of endogenous Cushing’s syndrome (SONICS): a Phase 3, multicentre, open-label, single-arm trial. Lancet Diabetes Endocrinol 7(11):855–865 10.1016/S2213-8587(19)30313-4 31542384

[39] Fleseriu M, Auchus RJ, Greenman Y, Zacharieva S, Geer EB, Salvatori R et al (2022) Levoketoconazole treatment in endogenous Cushing’s syndrome: extended evaluation of clinical, biochemical, and radiologic outcomes. Eur J Endocrinol 187(6):859–871 10.1530/EJE-22-0506 PMC9716395 36251618

[40] Viecceli C, Mattos ACV, Costa MCB, de Melo RB, Rodrigues TDC, Czepielewski MA (2022) Evaluation of ketoconazole as a treatment for Cushing’s disease in a retrospective cohort. Front Endocrinol (Lausanne) 13:1017331 10.3389/fendo.2022.1017331 PMC9585352 36277689

[41] Nieman LK, Boscaro M, Scaroni CM, Deutschbein T, Mezosi E, Driessens N et al (2021) Metyrapone treatment in endogenous cushing’s syndrome: results at week 12 from PROMPT, a prospective international multicenter, open-label, Phase III/IV study. J Endocr Soc 5(Suppl 1):A515

[42] Nieman L, Boscaro M, Carla S, Deutschbein T, Mezosi E, Driessens N et al (2021) Metyrapone treatment in endogenous Cushing’s syndrome. Long term efficacy and safety results of the extension of the Phase III/IV study PROMPT. Endocr Abstracts 73:abst OC3.3

[43] Colao A, Petersenn S, Newell-Price J, Findling JW, Gu F, Maldonado M et al (2012) A 12-month Phase 3 study of pasireotide in Cushing’s disease. N Engl J Med 366(10):914–924 10.1056/NEJMoa1105743 22397653

[44] Lacroix A, Gu F, Gallardo W, Pivonello R, Yu Y, Witek P et al (2018) Efficacy and safety of once-monthly pasireotide in Cushing’s disease: a 12 month clinical trial. Lancet Diabetes Endocrinol 6(1):17–26 10.1016/S2213-8587(17)30326-1 29032078

[45] Feelders RA, Fleseriu M, Kadioglu P, Bex M, González-Devia D, Boguszewski CL et al (2023) Long-term efficacy and safety of subcutaneous pasireotide alone or in combination with cabergoline in Cushing’s disease. Front Endocrinol (Lausanne) 14:1165681 10.3389/fendo.2023.1165681 PMC10593462 37876540

[46] Fleseriu M, Petersenn S, Biller BMK, Kadioglu P, De Block C, T’Sjoen G et al (2019) Long-term efficacy and safety of once-monthly pasireotide in Cushing’s disease: a Phase III extension study. Clin Endocrinol (Oxf) 91(6):776–785 10.1111/cen.14081 PMC6899900 31465533

[47] Gadelha MR, Gu F, Bronstein MD, Brue TC, Fleseriu M, Shimon I et al (2020) Risk factors and management of pasireotide-associated hyperglycemia in acromegaly. Endocr Connect 9(12):1178–1190 10.1530/EC-20-0361 PMC7774766 33434154

[48] Samson SL, Gu F, Feldt-Rasmussen U, Zhang S, Yu Y, Witek P et al (2021) Managing pasireotide-associated hyperglycemia: a randomized, open-label, Phase IV study. Pituitary 24(6):887–903 10.1007/s11102-021-01161-4 PMC8550309 34275099

[49] Fleseriu M, Biller BMK, Findling JW, Molitch ME, Schteingart DE, Gross C et al (2012) Mifepristone, a glucocorticoid receptor antagonist, produces clinical and metabolic benefits in patients with Cushing’s syndrome. J Clin Endocrinol Metab 97(6):2039–2049 10.1210/jc.2011-3350 22466348

[50] Whelton PK, Carey RM, Aronow WS, Casey DE, Collins KJ, Dennison C Himmelfarb et al (2018) 2017 ACC/AHA/AAPA/ABC/ACPM/AGS/APhA/ASH/ASPC/NMA/PCNA guideline for the prevention, detection, evaluation, and management of high blood pressure in adults: a report of the American College of Cardiology/American Heart Association Task Force on Clinical Practice Guidelines. Hypertension 71(6):e13–e115 10.1161/HYP.0000000000000065 29133356

[51] McEvoy JW, McCarthy CP, Bruno RM, Brouwers S, Canavan MD, Ceconi C et al (2024) 2024 ESC guidelines for the management of elevated blood pressure and hypertension. Eur Heart J 45(38):3912–4018 10.1093/eurheartj/ehae178 39210715

[52] Pivonello R, Zacharieva S, Elenkova A, Tóth M, Shimon I, Stigliano A et al (2022) Levoketoconazole in the treatment of patients with endogenous Cushing’s syndrome: a double-blind, placebo-controlled, randomized withdrawal study (LOGICS). Pituitary 25(6):911–926 10.1007/s11102-022-01263-7 PMC9675660 36085339

[53] Fleseriu M, Biller BM, Findling JW, Molitch ME, Schteingart DE, Gross C et al (2012) Mifepristone, a glucocorticoid receptor antagonist, produces clinical and metabolic benefits in patients with Cushing’s syndrome. J Clin Endocrinol Metab 97(6):2039–2049 10.1210/jc.2011-3350 22466348

