# Correction to: Osilodrostat improves blood pressure and glycemic control in patients with Cushing’s disease: a pooled analysis of LINC 3 and LINC 4 studies

**DOI:** 10.1007/s11102-025-01525-0

**Published:** 2025-05-21

**Authors:** Maria Fleseriu, Rosario Pivonello, John Newell-Price, Mônica R. Gadelha, Beverly M. K. Biller, Richard J. Auchus, Richard A. Feelders, Akira Shimatsu, Przemysław Witek, Marie Bex, Andrea Piacentini, Alberto M. Pedroncelli, André Lacroix

**Affiliations:** 1 Pituitary Center, Departments of Medicine and Neurological Surgery, Oregon Health and Science University, Portland, OR USA; 2 Dipartimento di Medicina Clinica e Chirurgia, Sezione di Endocrinologia, Università Federico II di Napoli, Naples, Italy; 3 School of Medicine and Population Health, University of Sheffield, Sheffield, UK; 4 Neuroendocrinology Research Center, Endocrinology Section, Medical School and Hospital Universitário Clementino Fraga Filho, Universidade Federal do Rio de Janeiro, Rio de Janeiro, Brazil; 5 Neuroendocrine and Pituitary Tumor Clinical Center, Massachusetts General Hospital, Boston, MA USA; 6 Department of Pharmacology, Division of Metabolism, Endocrinology and Diabetes, University of Michigan, Ann Arbor, MI USA; 7 Department of Internal Medicine, Endocrine Section, Erasmus Medical Center, Rotterdam, Netherlands; 8 Omi Medical Center, Kusatsu, Japan; 9 Department of Internal Medicine, Endocrinology and Diabetes, Medical University of Warsaw, Warsaw, Poland; 10 Department of Endocrinology, University Hospitals Leuven, Leuven, Belgium; 11 Recordati SpA, Milan, Italy; 12 Recordati AG, Basel, Switzerland; 13 Centre hospitalier de l’Université de Montréal, Montreal, Canada; 14 Present Address: Camurus AB, Lund, Sweden

**Pituitary (2025) 28:22**

**https://doi.org/10.1007/s11102-024-01471-3**

In the original publication of the article, the authors have found two errors in Fig. 2, as follows:

- In Fig. 2C, the definition of diabetes based on FPG levels in the figure key is currently ≤ 126 mg/dL (≤ 7.0 mmol/L); however, this should be ≥ 126 mg/dL (≥ 7.0 mmol/L).
- In Fig. 2D, the definition of normal HbA_1c_ levels in the figure key is currently ≥ 47.5 mmol/mol; however, this should be < 47.5 mmol/mol.

**Fig. 2:**
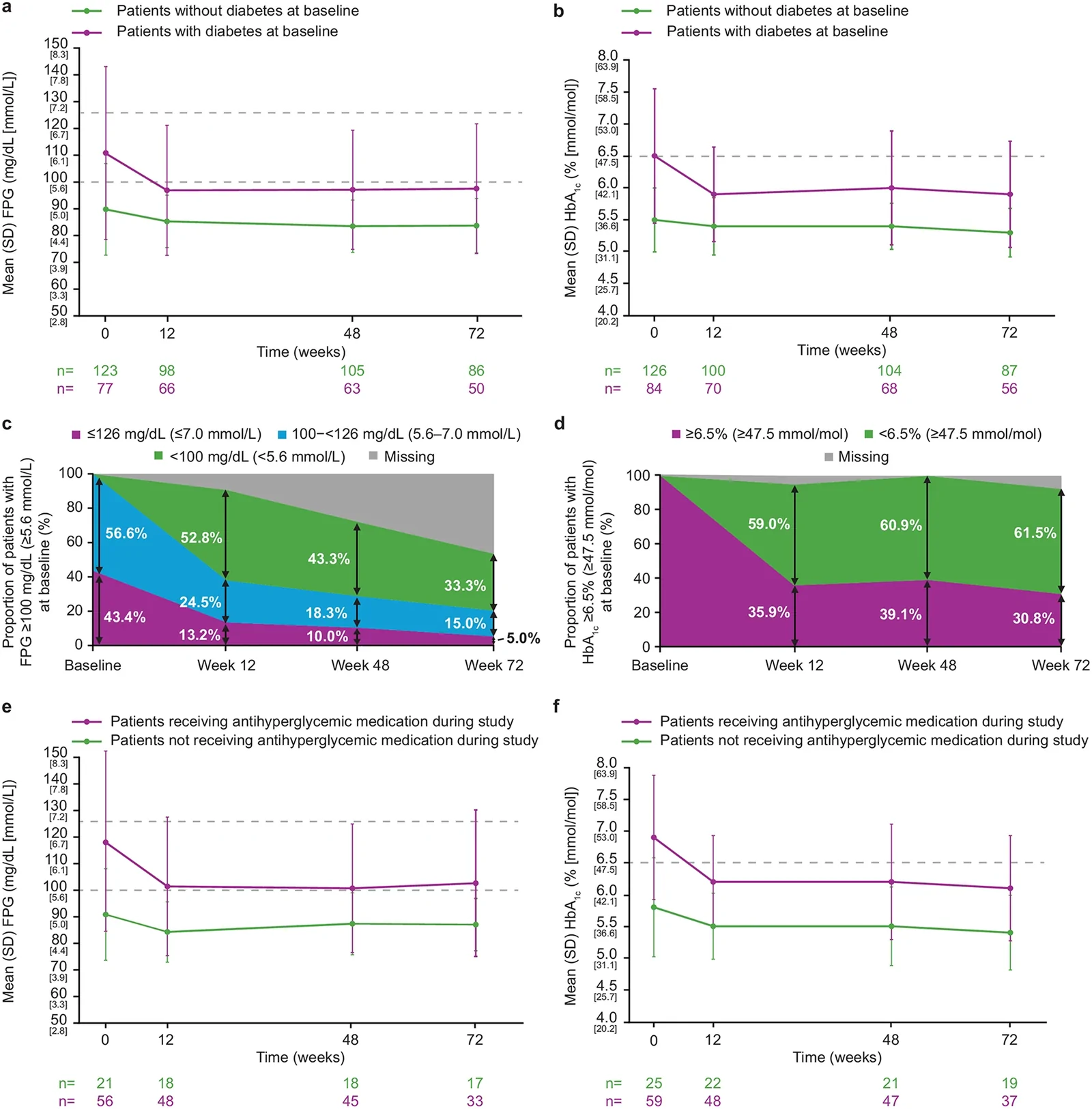
Mean (**a**) FPG and (**b**) HbA_1c_ over time in patients with and without diabetes at baseline. Shift over time in (**c**) FPG in patients with high FPG (≥ 100 mg/dL) at baseline and (**d**) HbA_1c_ in patients with high HbA_1c_ (≥ 6.5%) at baseline. Mean (**e**) FPG and (**f**) HbA_1c_ over time in patients with diabetes at baseline, according to antihyperglycemic medication use during the studies. For panels a and e, the dashed gray lines indicate the FPG thresholds for pre-diabetes (100 mg/dL [5.6 mmol/L]) and diabetes (126 mg/dL [7.0 mmol/L]). For panels b and f, the dashed gray lines indicate the HbA_1c_ threshold for diabetes (6.5% [47.5 mmol/mol])

Hence, the corrected Fig. 2 is given below:

**Fig. 2:**
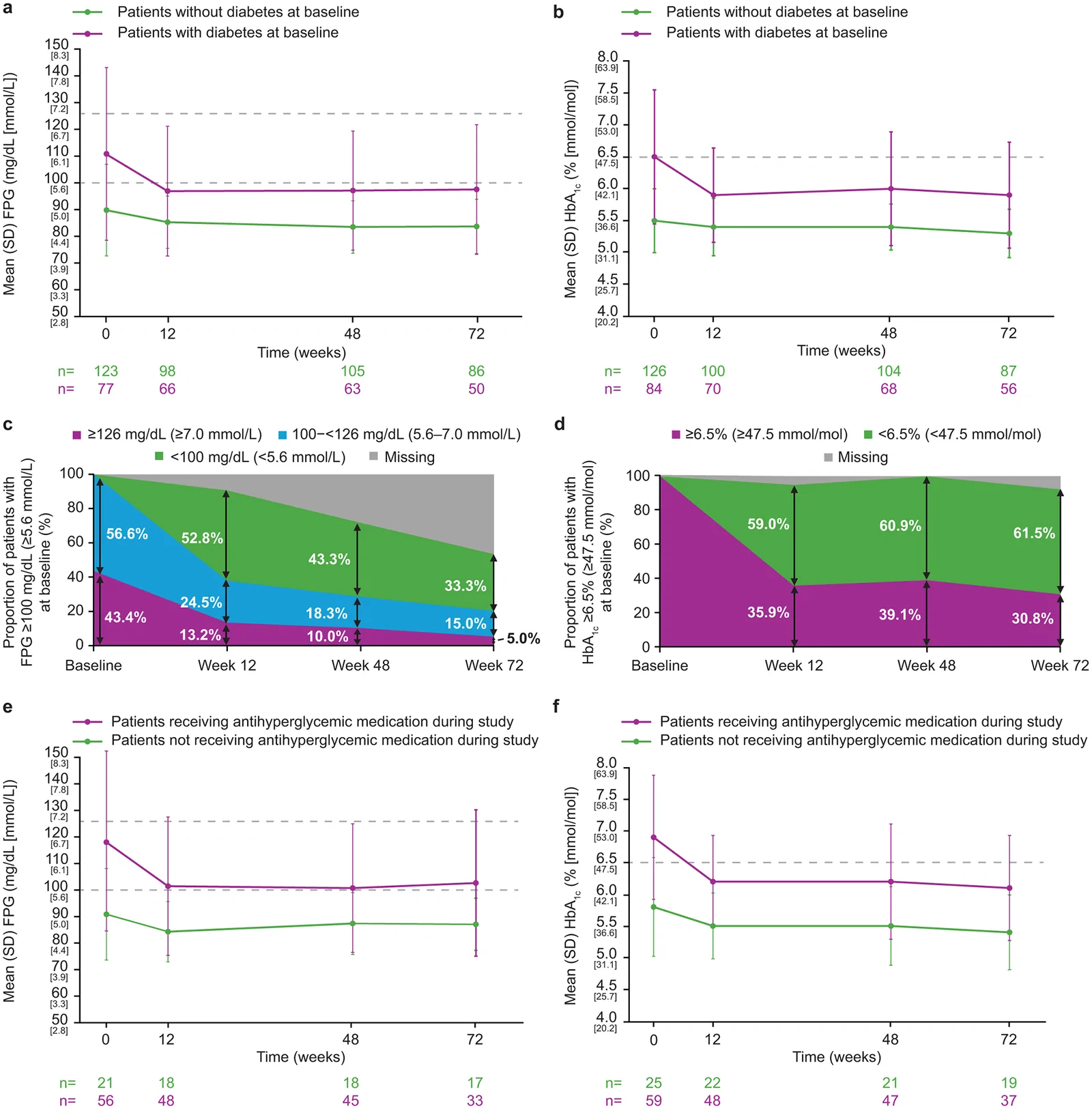
Mean (**a**) FPG and (**b**) HbA_1c_ over time in patients with and without diabetes at baseline. Shift over time in (**c**) FPG in patients with high FPG (≥ 100 mg/dL) at baseline and (**d**) HbA_1c_ in patients with high HbA_1c_ (≥ 6.5%) at baseline. Mean (**e**) FPG and (**f**) HbA_1c_ over time in patients with diabetes at baseline, according to antihyperglycemic medication use during the studies. For panels a and e, the dashed gray lines indicate the FPG thresholds for pre-diabetes (100 mg/dL [5.6 mmol/L]) and diabetes (126 mg/dL [7.0 mmol/L]). For panels b and f, the dashed gray lines indicate the HbA_1c_ threshold for diabetes (6.5% [47.5 mmol/mol])

The original article has been corrected.

