# Supplementary material for: Osilodrostat improves blood pressure and glycemic control in patients with Cushing’s disease: a pooled analysis of LINC 3 and LINC 4 studies
Source: Pituitary. 2025 Jan 25;28(1):22. doi: 10.1007/s11102-024-01471-3 (PMC11762609; doi:10.1007/s11102-024-01471-3)

Osilodrostat improves blood pressure and glycemic control in patients with Cushing’s disease: a pooled analysis of LINC 3 and LINC 4 studies

Supplementary information

[Supplementary Table 1:](#ST1) Patient demographics and baseline characteristics for patients with neither hypertension nor diabetes, or with both, at baseline and for patients with only one of the two comorbidities

[Supplementary Figure 1:](#SF1) Correlation between baseline SBP and change in SBP from baseline to a) W12 and b) W72, and between baseline DBP and change in DBP from baseline to c) W12 and d) W72

[Supplementary Figure 2:](#SF2) Mean a) 11-deoxycorticosterone and b) 11-deoxycortisol levels in patients with and without hypertension at baseline

[Supplementary Figure 3:](#SF3) Mean a) 11-deoxycorticosterone and b) 11-deoxycortisol levels over time in patients with and without hypokalemia at baseline

[Supplementary Figure 4:](#SF4) Mean potassium levels in patients with and without hypertension at baseline

[Supplementary Figure 5:](#SF5) Mean SBP and DBP over time according to changes in antihypertensive medication use during the studies

[Supplementary Figure 6:](#SF6) Mean SBP and DBP over time, by baseline mUFC severity, in patients with hypertension at baseline

[Supplementary Figure 7:](#SF7) Mean SBP and DBP over time in patients with hypertension at baseline, by mUFC control, at a) W12, b) W48, and c) W72

[Supplementary Figure 8](#SF8): Changes in antihypertensive medication use according to mUFC control in a) patients with hypertension at baseline and taking antihypertensive medication and b) patients without hypertension who started antihypertensive medication during the studies

[Supplementary Figure 9:](#SF9) Change in spironolactone use according to mUFC control in patients taking spironolactone at baseline

[Supplementary Figure 10:](#SF10) Mean a) weight, b) waist circumference, and c) BMI over time, by presence or absence of hypertension at baseline

[Supplementary Figure 11:](#SF11) Correlation between baseline FPG and change in FPG from baseline to a) W12 and b) W72, and between baseline HbA_1c_ and change in HbA_1c_ from baseline to c) W12 and d) W72

[Supplementary Figure 12:](#SF12) Mean a) FPG and b) HbA_1c_ over time, according to changes in antihyperglycemic medication use during the studies

[Supplementary Figure 13:](#SF14) Mean FPG levels at a) W12, b) W48, and c) W72, and mean HbA_1c_ levels at d) W12, e) W48, and f) W72, over time in patients with diabetes at baseline, by mUFC control

[Supplementary Figure 14:](#SF15) Changes in antihyperglycemic medication use according to mUFC control in a) patients with diabetes at baseline and taking antihyperglycemic medication and b) patients without diabetes who started antihyperglycemic medication during the studies

[Supplementary Figure 15:](#SF16) Mean a) weight, b) waist circumference, and c) BMI over time, by presence/absence of diabetes at baseline

Supplementary Table 1. Patient demographics and baseline characteristics for patients with neither hypertension nor diabetes, or with both, at baseline and for patients with only one of the two comorbidities

|  | Patients without hypertension or diabetes  N=27 | Patients with diabetes but not hypertension  N=9 | Patients with hypertension but not diabetes  N=99 | Patients with both hypertension and diabetes  N=75 |
| --- | --- | --- | --- | --- |
| Median age, years (min–max) | 32.0 (19–49) | 40.0 (22–55) | 40.0 (19–70) | 44.0 (19–69) |
| Sex, n (%)  Female  Male | 25 (92.6)  2 (7.4) | 8 (88.9)  1 (11.1) | 73 (73.7)  26 (26.3) | 61 (81.3)  14 (18.7) |
| Race, n (%)  Caucasian  Asian  Black  Native American  Other  Unknown | 18 (66.7)  8 (29.6)  0  0  1 (3.7)  0 | 6 (66.7)  1 (11.1)  0  0  1 (11.1)  1 (11.1) | 65 (65.7)  27 (27.3)  4 (4.0)  0  2 (2.0)  1 (1.0) | 49 (65.3)  20 (26.7)  2 (2.7)  1 (1.3)  2 (2.7)  1 (1.3) |
| Weight, kg  Mean (SD)  Median (min–max) | 71.9 (17.4)  67.2 (47–112) | 89.3 (20.9)  92.5 (56–118) | 78.7 (20.2)  75.4 (46–165) | 83.2 (21.9)  74.7 (48–142) |
| Waist circumference, cm^a^  Mean (SD)  Median (min–max) | 95.9 (18.0)  95.0 (67–144) | 116.7 (27.6)  109.0 (91–171) | 100.8 (18.0)  98.0 (67–158) | 107.4 (15.9)  104.5 (78–151) |
| BMI, kg/m^2^  Mean (SD)  Median (min–max) | 27.5 (6.2)  27.4 (19–45) | 32.7 (8.9)  34.3 (24–51) | 29.5 (6.8)  28.7 (18–51) | 31.7 (7.7)  29.9 (20–56) |
| Median time to first osilodrostat dose since diagnosis, months (min–max) | 70.4 (4–241) | 48.1 (6–278) | 63.0 (2–240) | 38.8 (3–287) |
| Disease status, n (%)  *De novo*  Persistent/recurrent | 1 (3.7)  26 (96.3) | 0  9 (100) | 9 (9.1)  90 (90.9) | 10 (13.3)  65 (86.7) |
| Proportion of patients with previous pituitary surgery, n (%) | 26 (96.3) | 9 (100) | 87 (87.9) | 62 (82.7) |
| Proportion of patients with any previous medical treatment for Cushing’s disease, n (%) | 21 (77.8) | 7 (77.8) | 84 (84.8) | 64 (85.3) |
| Proportion of patients with previous pituitary irradiation, n (%) | 5 (18.5) | 0 | 13 (13.1) | 13 (17.3) |
| Proportion of patients with comorbidities, n (%) | 27 (100) | 9 (100) | 99 (100) | 75 (100) |
| Mean number of comorbidities (SD) | 5.9 (4.8) | 8.4 (4.4) | 9.0 (5.9) | 13.2 (10.6) |
| mUFC, nmol/24 h  Mean (SD), nmol/24 h  Mean (SD), µg/24 h  Median (min–max), nmol/24 h  Median (min–max), µg/24 h | 662.7 (1178.8)  240.2 (427.3) 4.8 (8.5) x ULN 321.7 (145–5720)  116.6 (52.6–2073.5) 2.3 (1.1–41.4) x ULN | 416.1 (259.1)  150.8 (93.9)  3.0 (1.9) x ULN  369.5 (67–781)  133.9 (24.3–283.1)  2.7 (0.5–5.7) x ULN | 775.7 (1247.0)  281.2 (452.0)  5.6 (9.0) x ULN  416.3 (47–9494)  150.9 (17–3441.6)  3.0 (0.3–68.8) x ULN | 945.4 (1550.2)  342.7 (561.9)  6.9 (11.2) x ULN  434.2 (21–9612)  157.4 (7.6–3484.4)  3.1 (0.2–69.7) x ULN |

ULN for mUFC is 138 nmol/24 h (50 μg/24 h). ^a^n=27, n=9, n=96, n=74, respectively. BMI, body mass index; mUFC, mean urinary free cortisol; SD, standard deviation; ULN, upper limit of normal

The largest decreases in systolic/diastolic blood pressure (SBP/DBP) occurred in patients with the highest baseline SBP/DBP, as shown by the negative correlation between baseline SBP/DBP and the corresponding changes from baseline at week (W)12 (r=−0.64 and r=−0.59, both *P*<0.0001) and W72 (r=−0.69 and r=−0.64, both *P*<0.0001; Supplementary Figure 1).

Supplementary Figure 1. Correlation between baseline SBP and change in SBP from baseline to a) W12 and b) W72, and between baseline DBP and change in DBP from baseline to c) W12 and d) W72

a)


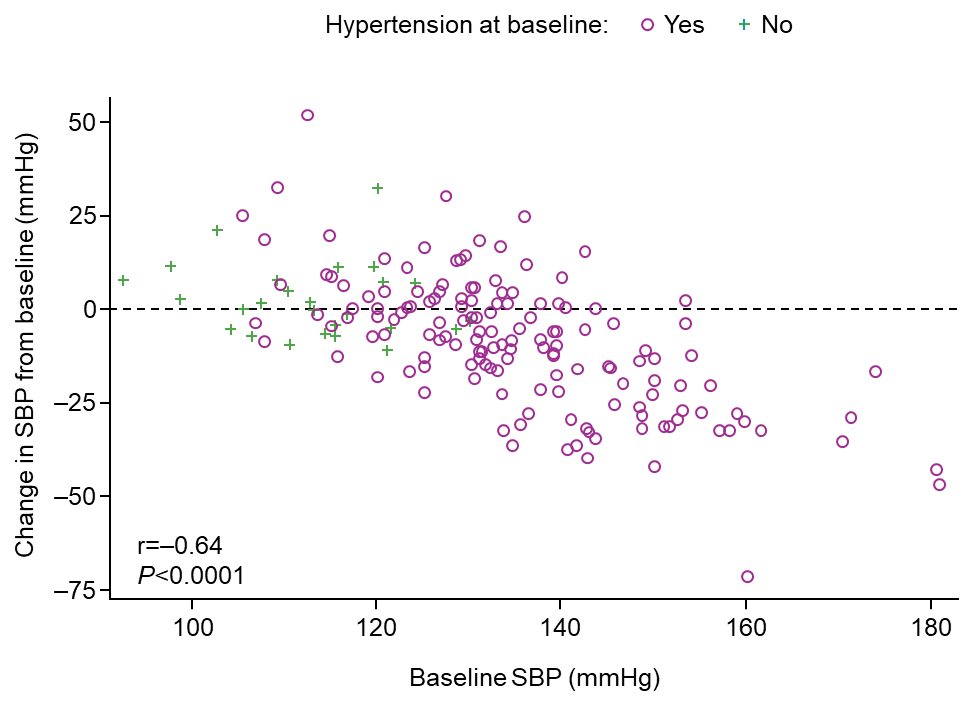


b)


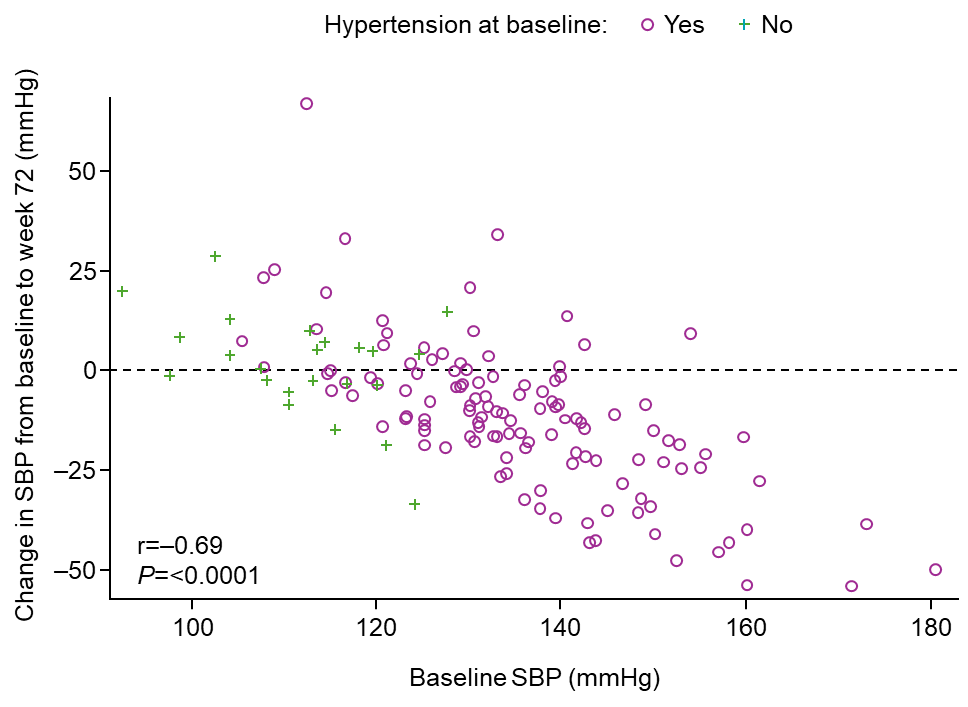


c)


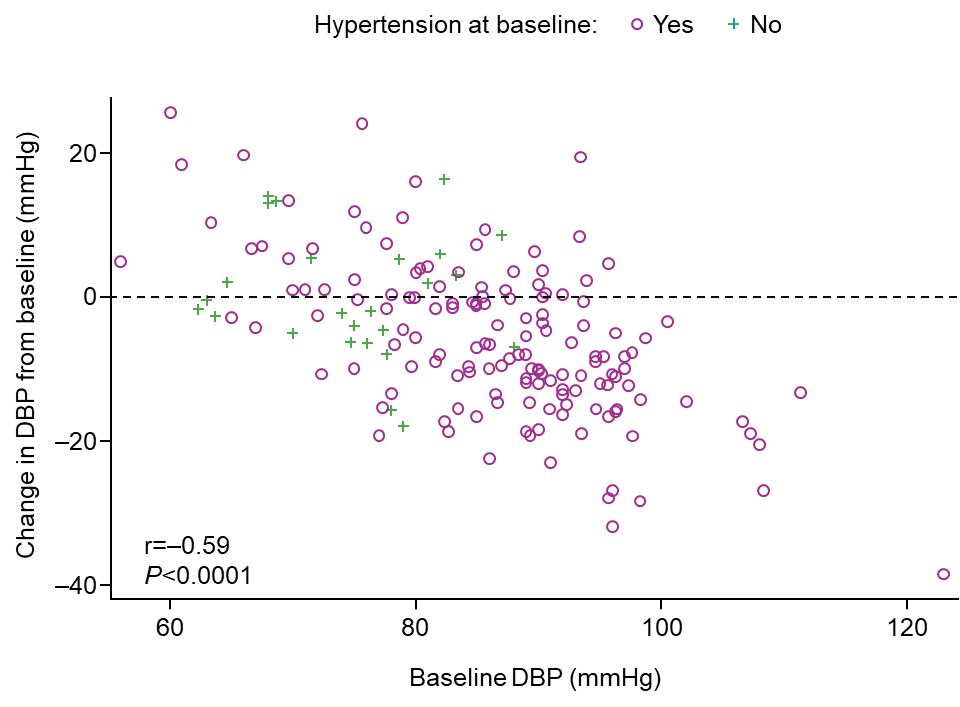


d)


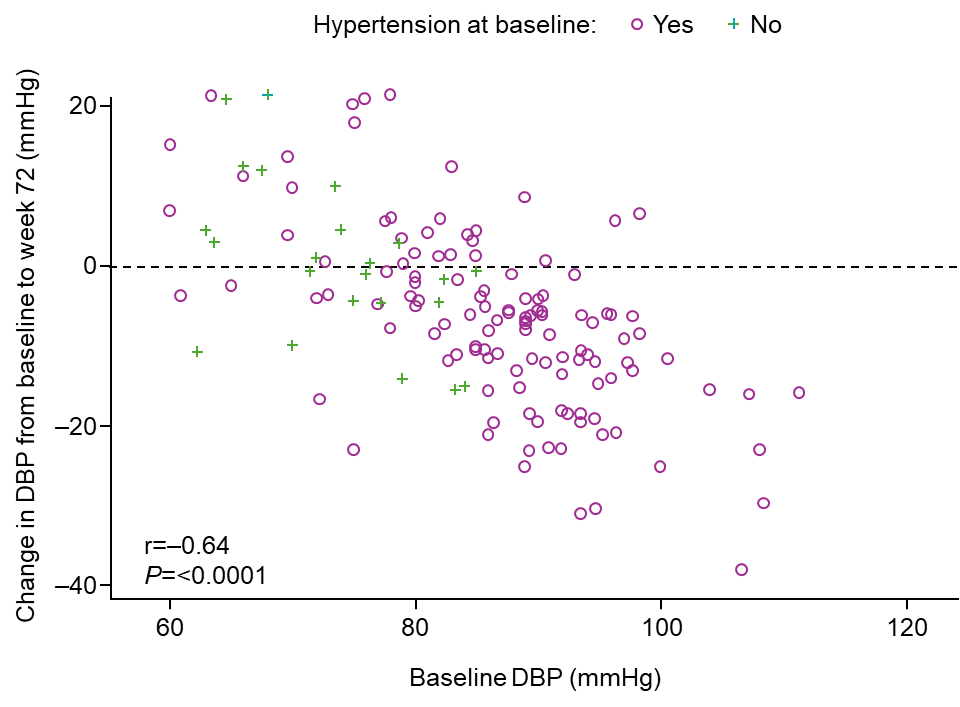


Mean levels of both 11-deoxycorticosterone and 11-deoxycortisol increased between baseline and W72 in patients with hypertension at baseline (Supplementary Figure 2). For patients without hypertension at baseline, a similar trend was observed for
11-deoxycortisol in patients without hypertension at baseline, but limited data prevent meaningful insight for 11-deoxycorticosterone.

Supplementary Figure 2. Mean a) 11-deoxycorticosterone and b) 11-deoxycortisol levels in patients with and without hypertension at baseline

a)


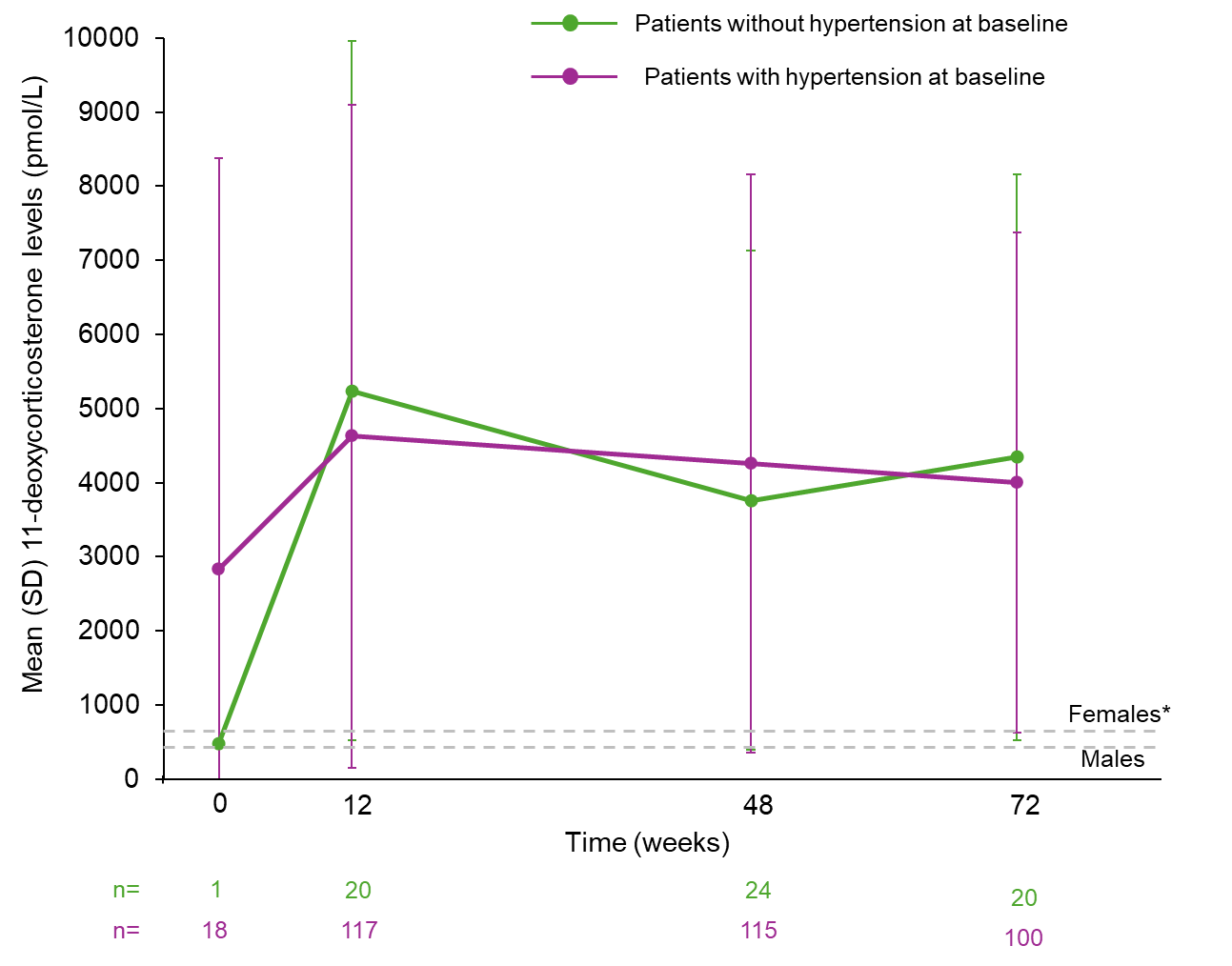


b)


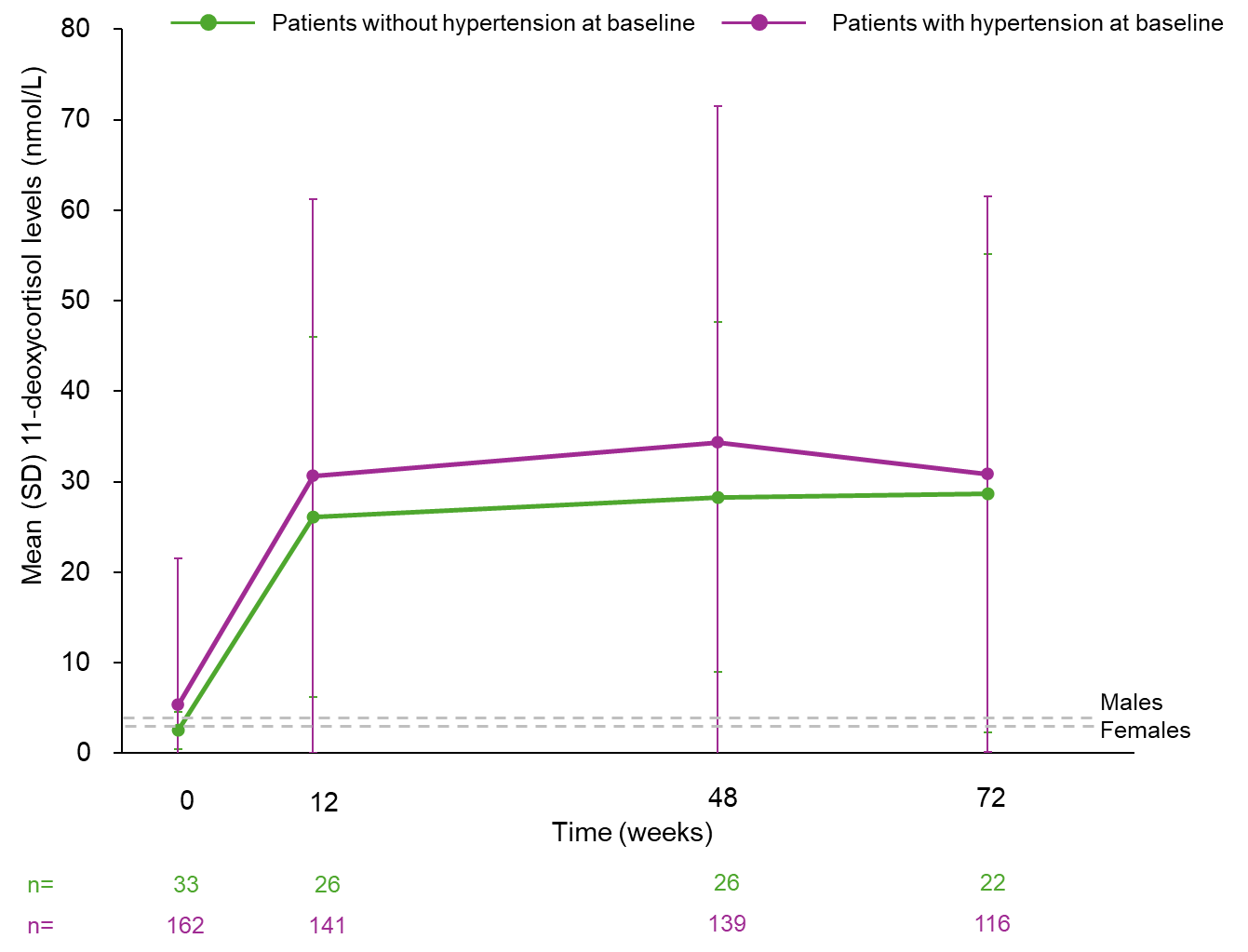


For panel a, dashed lines represent the ULN for 11-deoxycorticosterone in males (455 pmol/L) and females (mid-cycle; 696 pmol/L). For panel b, dashed lines represent the ULN for 11-deoxycorticosterol in males (3.9 nmol/L) and females (3.1 nmol/L)

Mean baseline levels of 11-deoxycorticosterone and 11-deoxycortisol were lower in patients without than with hypokalemia at baseline; by W72, mean levels of both were similar in those with and without hypokalemia (Supplementary Figure 3). However, the small number of patients without hypokalemia at baseline limits the interpretation of these data.

Supplementary Figure 3. Mean a) 11-deoxycorticosterone and b) 11-deoxycortisol levels over time in patients with and without hypokalemia at baseline

a)


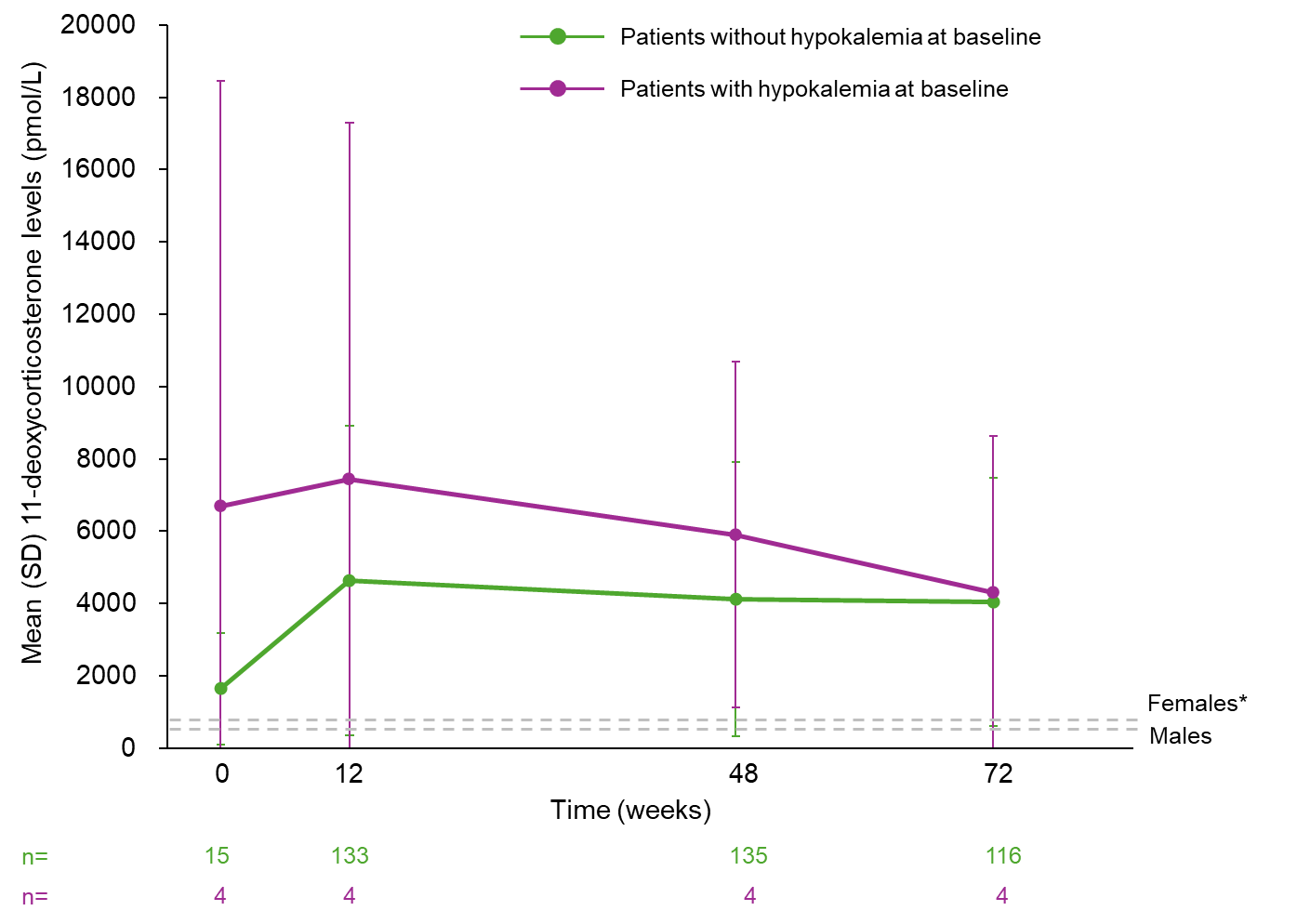


b)


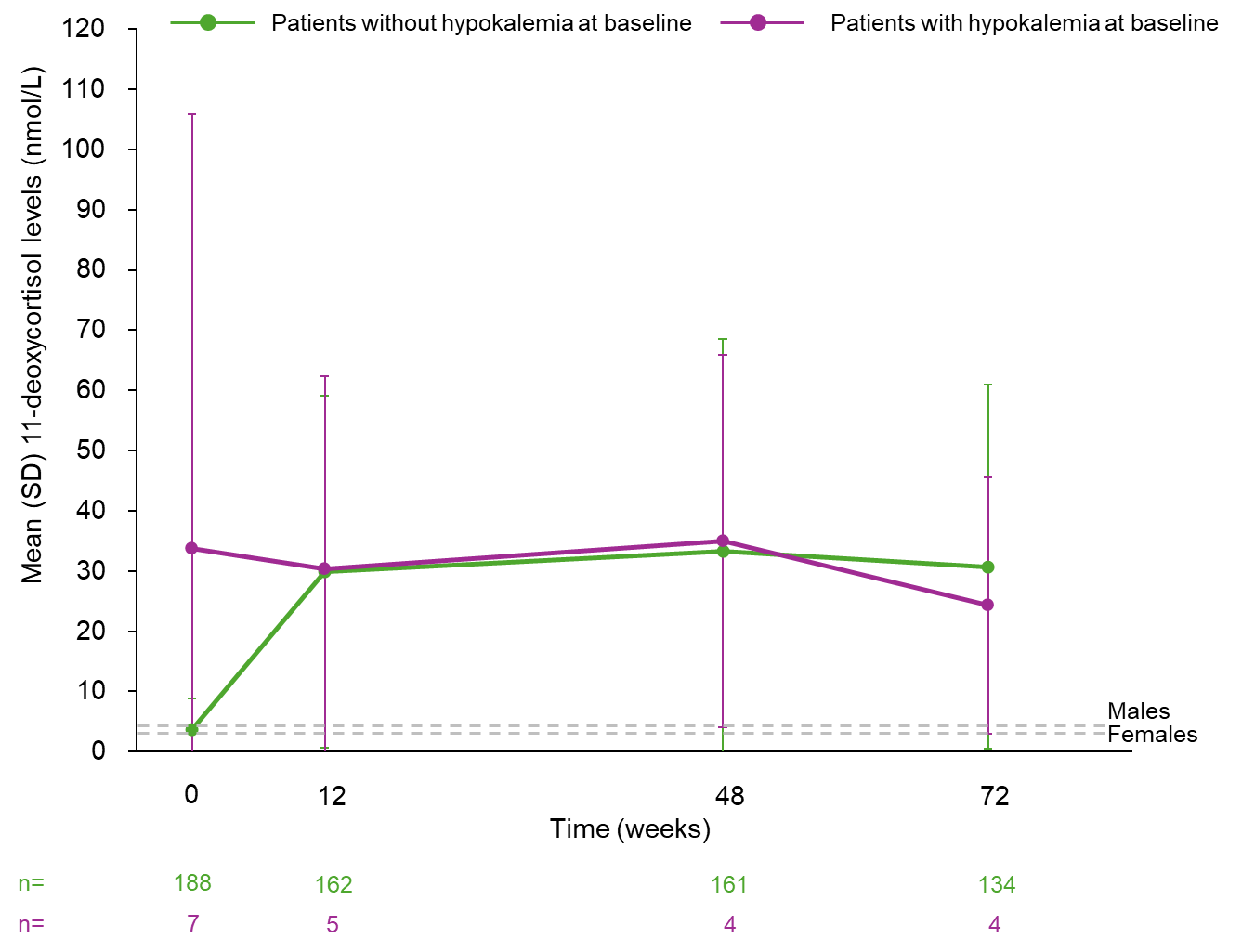


For panel a, dashed lines represent the ULN for 11-deoxycorticosterone in males (455 pmol/L) and females (mid-cycle; 696 pmol/L). For panel b, dashed lines represent the ULN for 11-deoxycorticosterol in males (3.9 nmol/L) and females (3.1 nmol/L)

Potassium levels remained within the normal range in patients with and without hypertension at baseline; mean (SD) levels were 4.1 (0.4) and 4.2 (0.4) mmol/L, respectively, at baseline and 4.1 (0.4) and 4.1 (0.3) mmol/L, respectively, at W72 (Supplementary Figure 4). Among 134 patients who had normal potassium levels at baseline, levels remained normal in 128 (95.5%) at W72 and were low in the remaining six (4.5%). Of the six patients with low potassium levels at baseline, all had normal levels at W72.

Supplementary Figure 4. Mean potassium levels in patients with and without hypertension at baseline


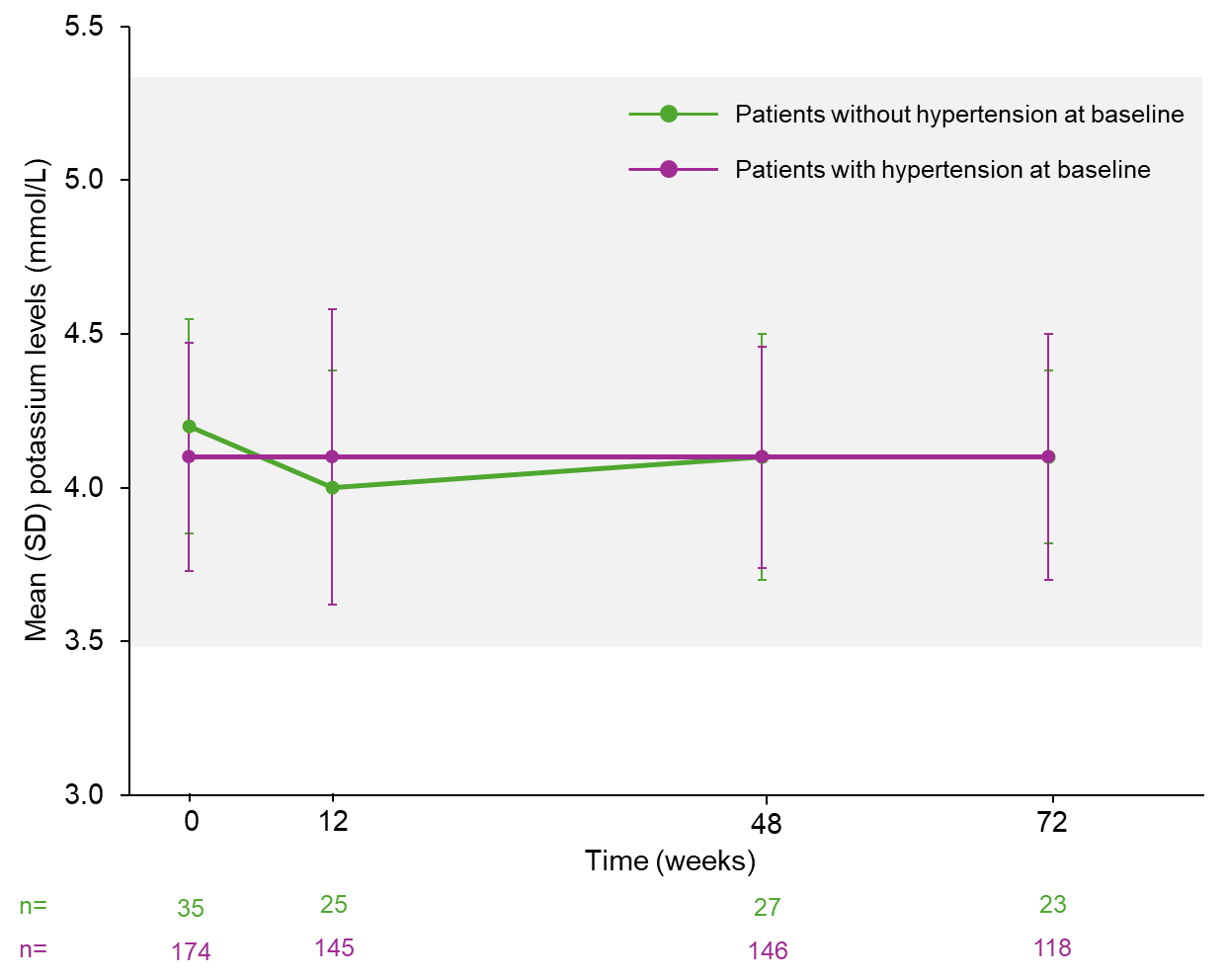


Gray region represents the normal range for potassium (3.5–5.3 mmol/L)

Supplementary Figure 5. Mean SBP and DBP over time according to changes in antihypertensive medication use during the studies


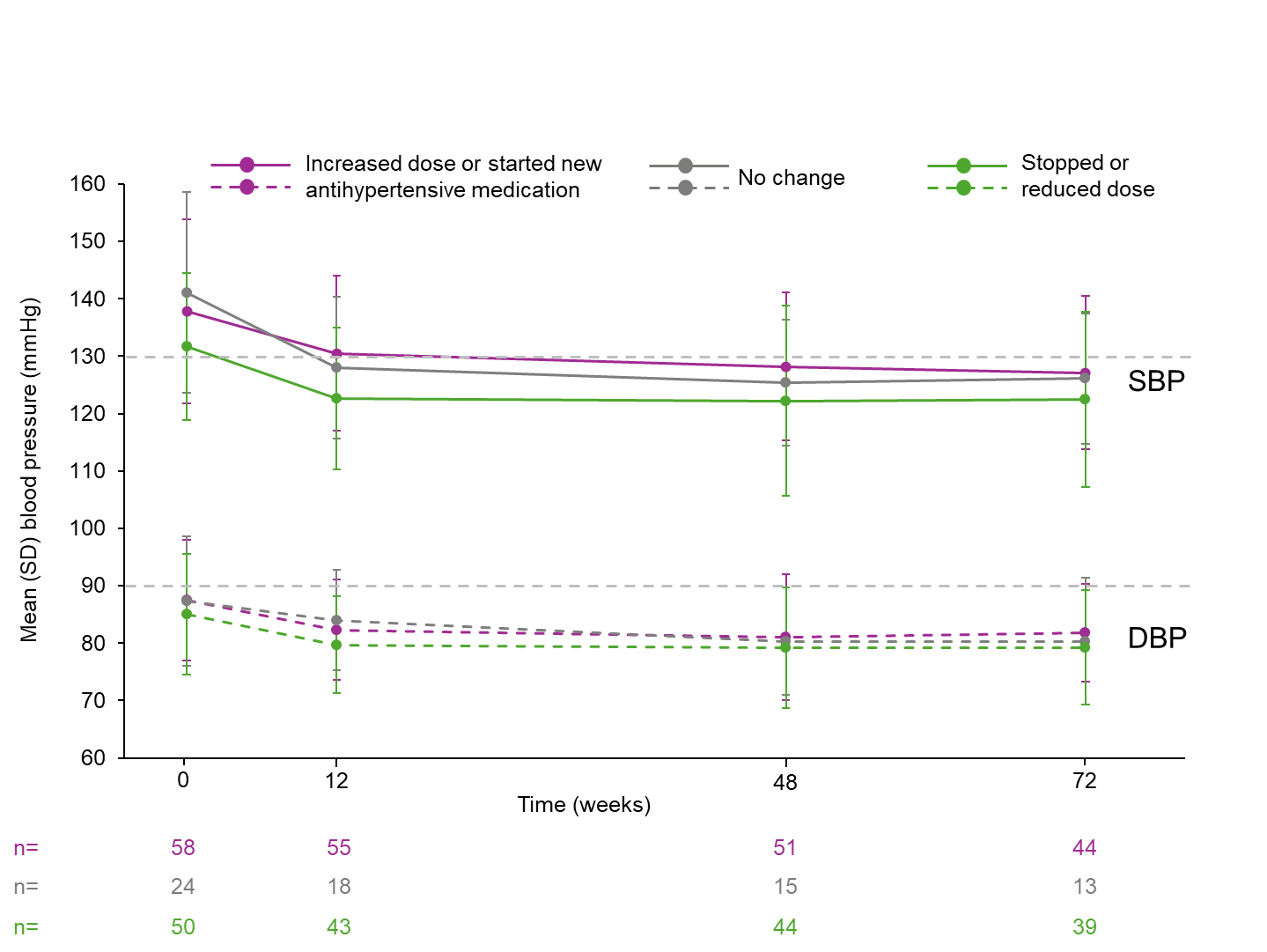


Dashed gray lines represent upper limits above which hypertension is indicated: SBP 130 mmHg and DBP 90 mmHg

Supplementary Figure 6. Mean SBP and DBP over time, by baseline mUFC severity, in patients with hypertension at baseline


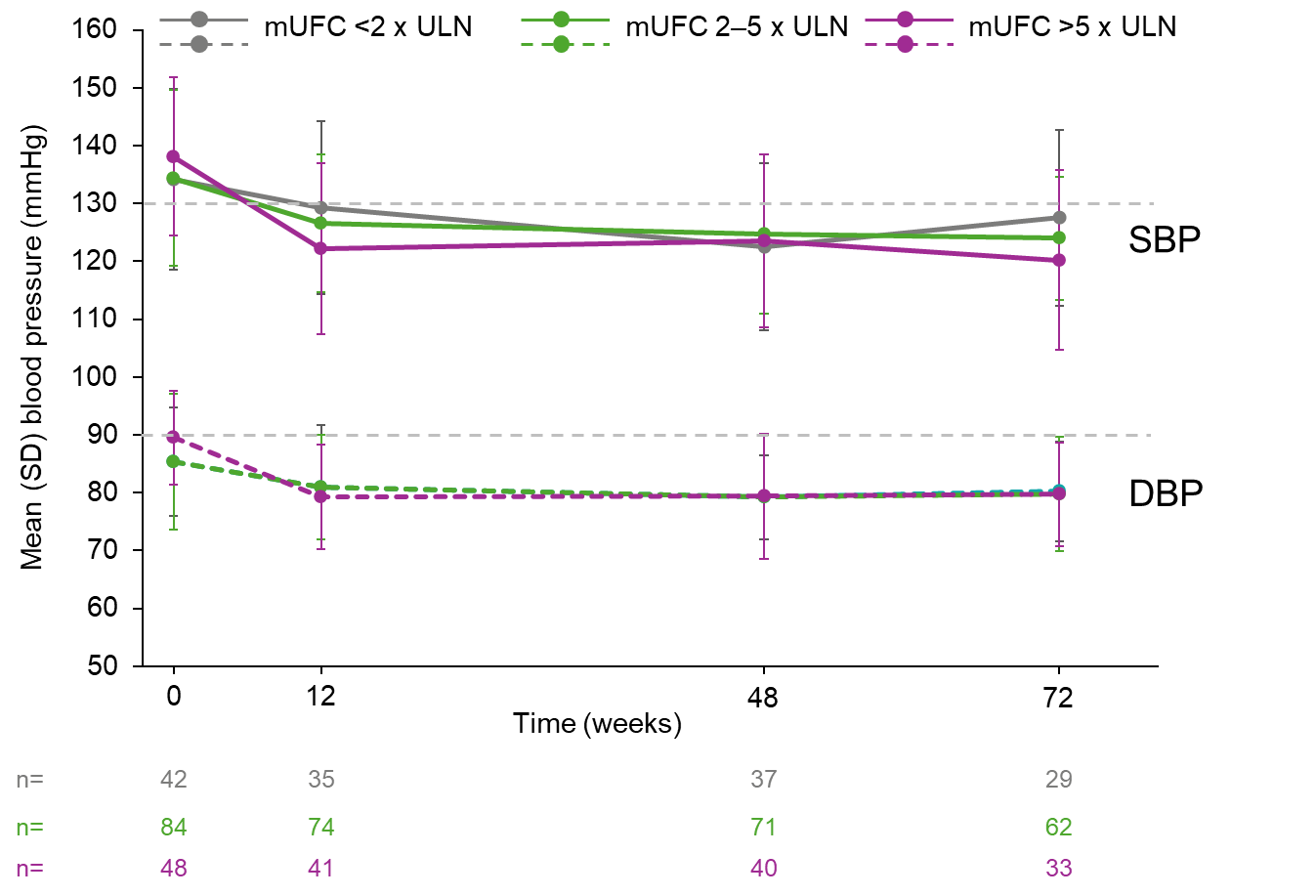

Dashed gray lines represent upper limits above which hypertension is indicated: SBP 130 mmHg and
DBP 90 mmHg

Supplementary Figure 7. Mean SBP and DBP over time in patients with hypertension at baseline, by mUFC control, at a) W12, b) W48, and c) W72


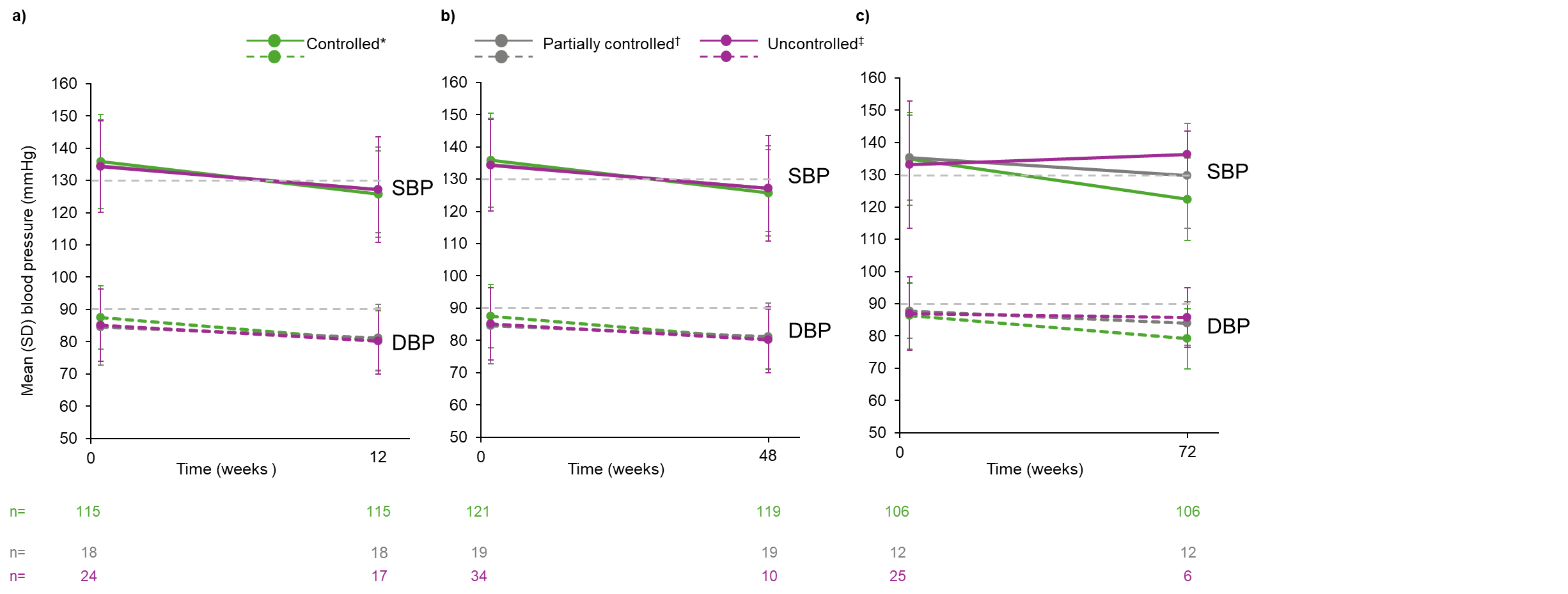


*mUFC ≤ULN; ^†^mUFC >ULN but ≥50% decrease from baseline; ^‡^mUFC >ULN and <50% decrease from baseline. Dashed gray lines represent upper limits above which hypertension is indicated: SBP 130 mmHg and DBP 90 mmHg

In patients with hypertension at baseline who used antihypertensive medication during the studies, the proportion who reduced or stopped their dose was higher in those with mUFC ≤ULN and partial mUFC control than in those with uncontrolled mUFC at W12 and W48, but not at W72 (Supplementary Figure 8).

Supplementary Figure 8. Changes in antihypertensive medication use according to mUFC control in patients with hypertension at baseline and taking antihypertensive medication


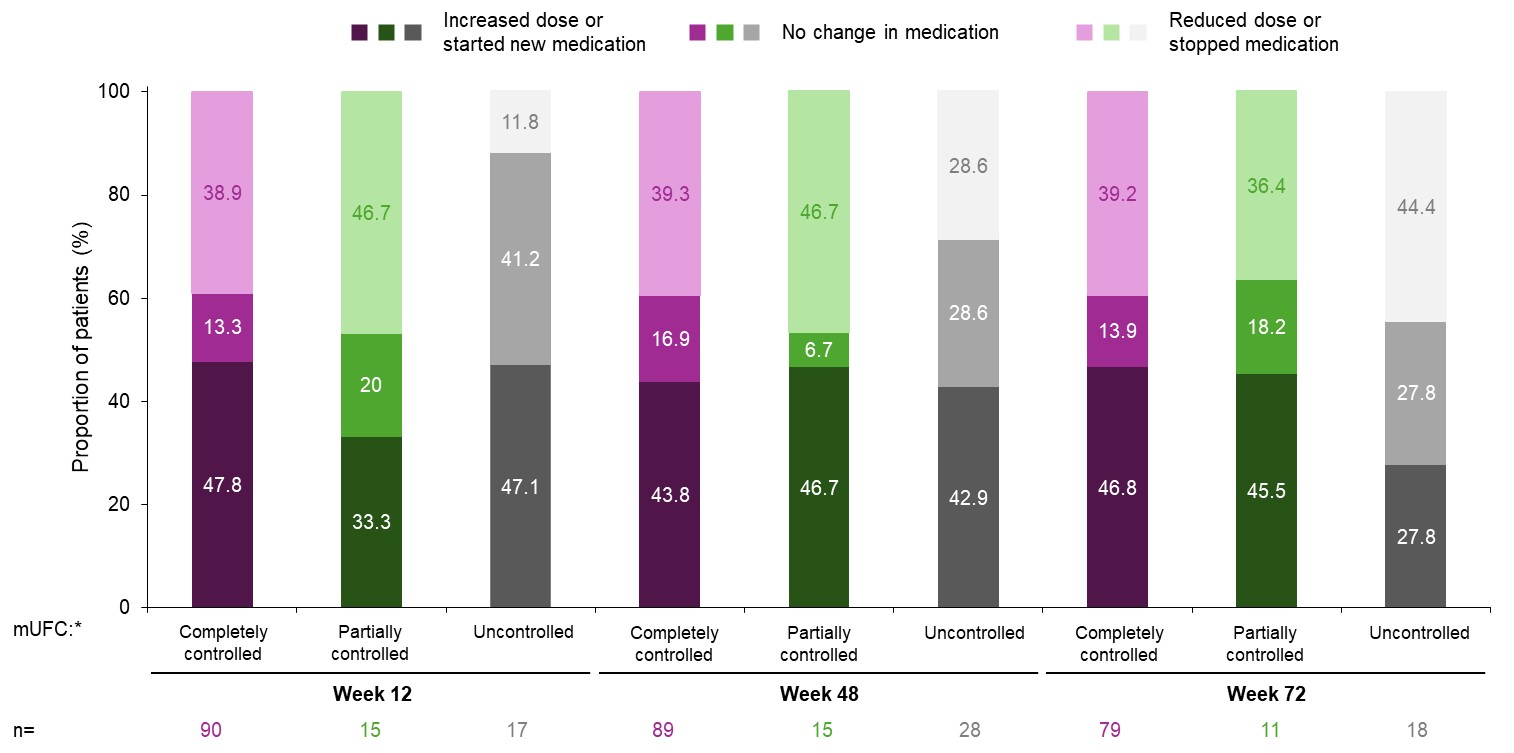


*Controlled: mUFC ≤ULN; partially controlled: mUFC >ULN but ≥50% decrease from baseline; uncontrolled: mUFC>ULN and <50% decrease from baseline

As only four patients without hypertension at baseline started antihypertensive medication during the studies, the change in medication according to mUFC control is not reported here.

Of 16 patients taking spironolactone at baseline, 12 stopped treatment by W72 (nine of these during the first 12 weeks). Among patients taking spironolactone at baseline, 66.7% of those who stopped treatment and 50.0% of those who continued treatment had mUFC ≤ULN at W12; corresponding values at W72 were 75.0% and 75.0%, respectively (Supplementary Figure 9).

Supplementary Figure 9. Change in spironolactone use according to mUFC control in patients taking spironolactone at baseline


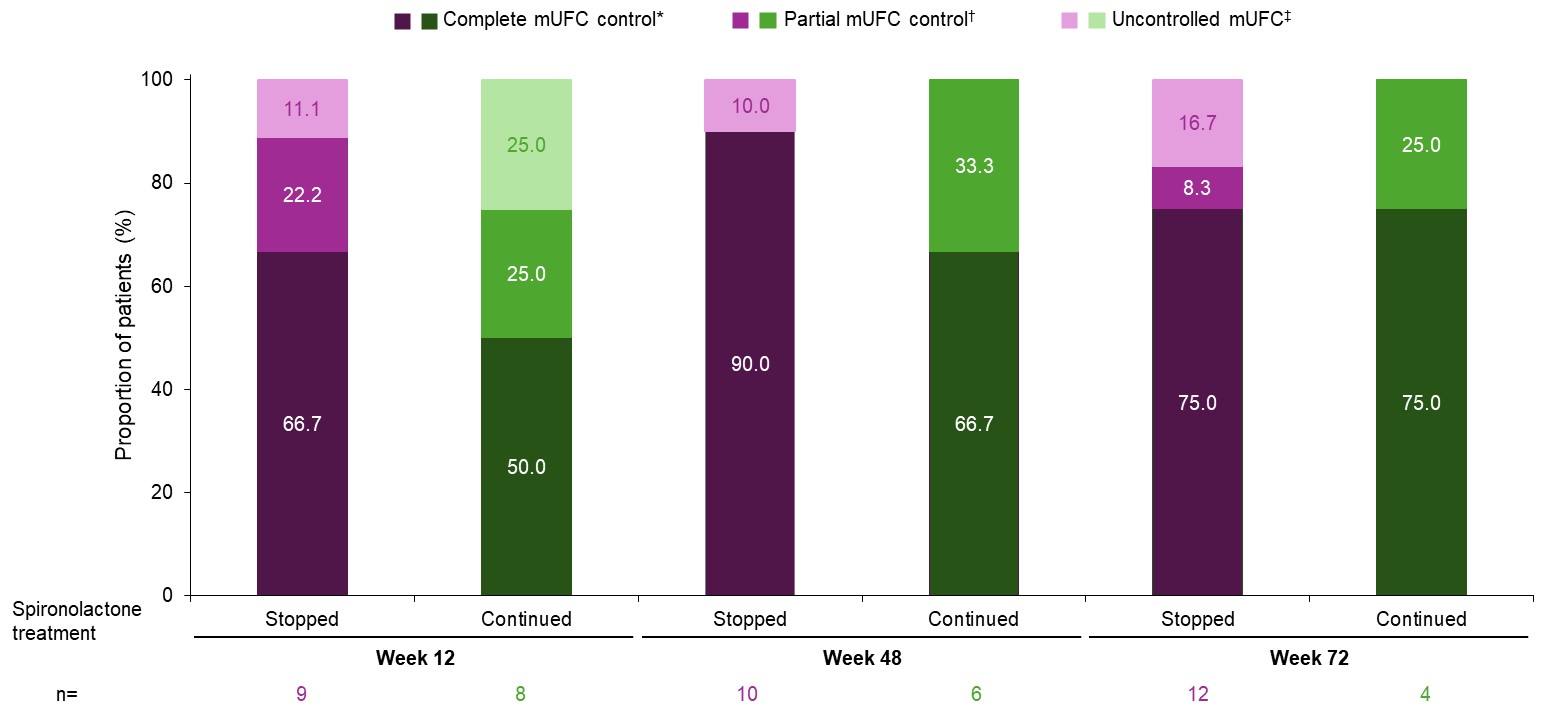


*mUFC ≤ULN; ^†^mUFC >ULN but ≥50% decrease from baseline; ^‡^mUFC>ULN and <50% decrease from baseline

Reductions in mean (SD) weight, waist circumference, and BMI were observed from baseline to W12 in patients with and without hypertension at baseline (respectively, –0.9 [3.3] and –0.5 [3.0] kg, –1.1 [6.4] and –1.4 [4.7] cm, –0.4 [1.3] and –0.2 [1.1] kg/m^2^); reductions were maintained over long-term treatment in both subgroups (Supplementary Figure 10). There was no correlation between change in SBP/DBP and change in weight from baseline to W72 in patients with (SBP: r=0.06, *P*=0.531; DBP: r=0.05, *P*=0.561) and without hypertension at baseline (SBP: r=0.34, *P*=0.114; DBP: r=0.31, *P*=0.153).

Supplementary Figure 10. Mean a) weight, b) waist circumference, and c) BMI over time, by presence/absence of hypertension at baseline


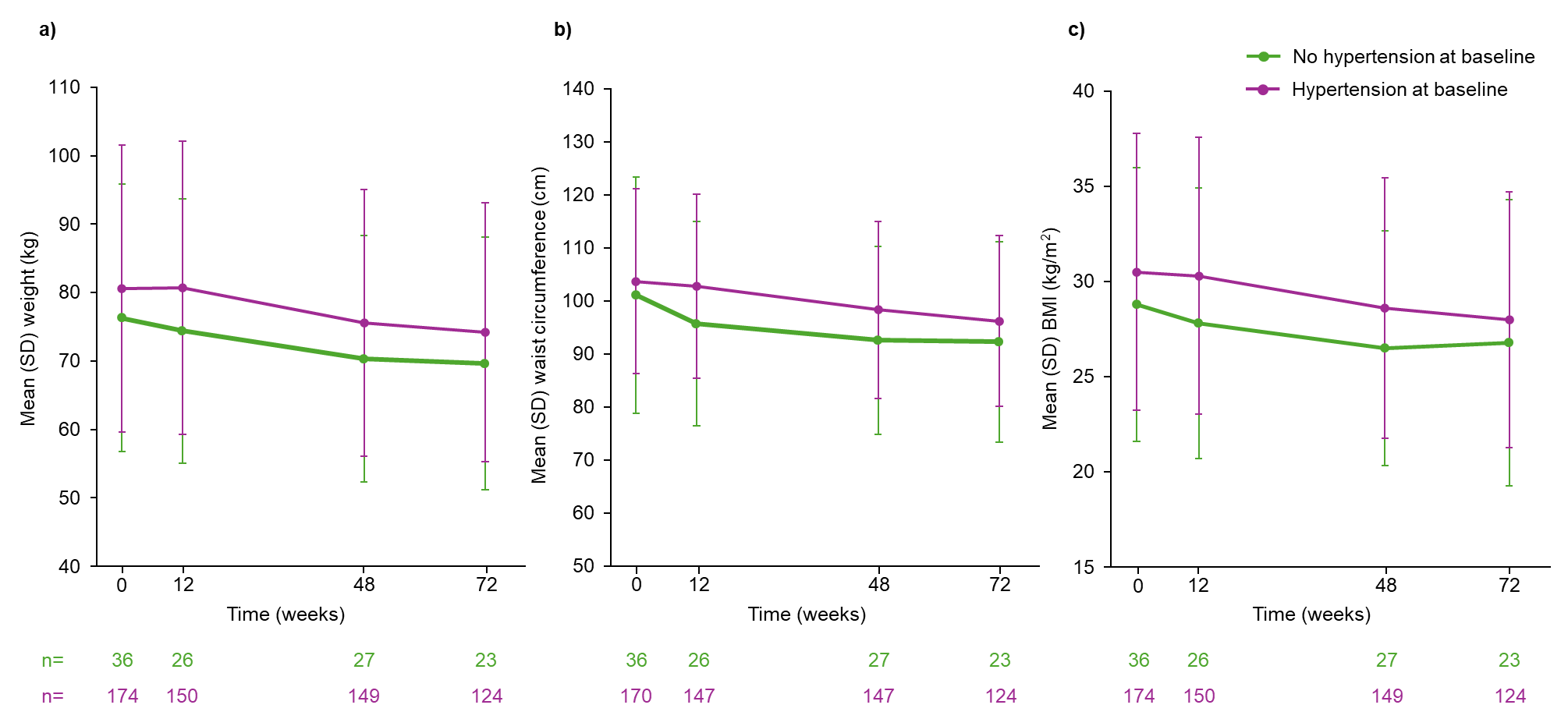


The largest decreases in fasting plasma glucose (FPG) and glycated hemoglobin (HbA_1c_) occurred in patients with the highest baseline values, as shown by the negative correlation between baseline FPG and HbA_1c_ and the corresponding changes from baseline to W12 (r=−0.77 and r=−0.75, both *P*<0.0001) and W72 (r=−0.60 and r=−0.68, both *P*<0.0001; Supplementary Figure 11).

Supplementary Figure 11. Correlation between baseline FPG and change in FPG from baseline to a) W12 and b) W72, and between baseline HbA_1c_ and change in HbA_1c_ from baseline to c) W12 and d) W72

a)


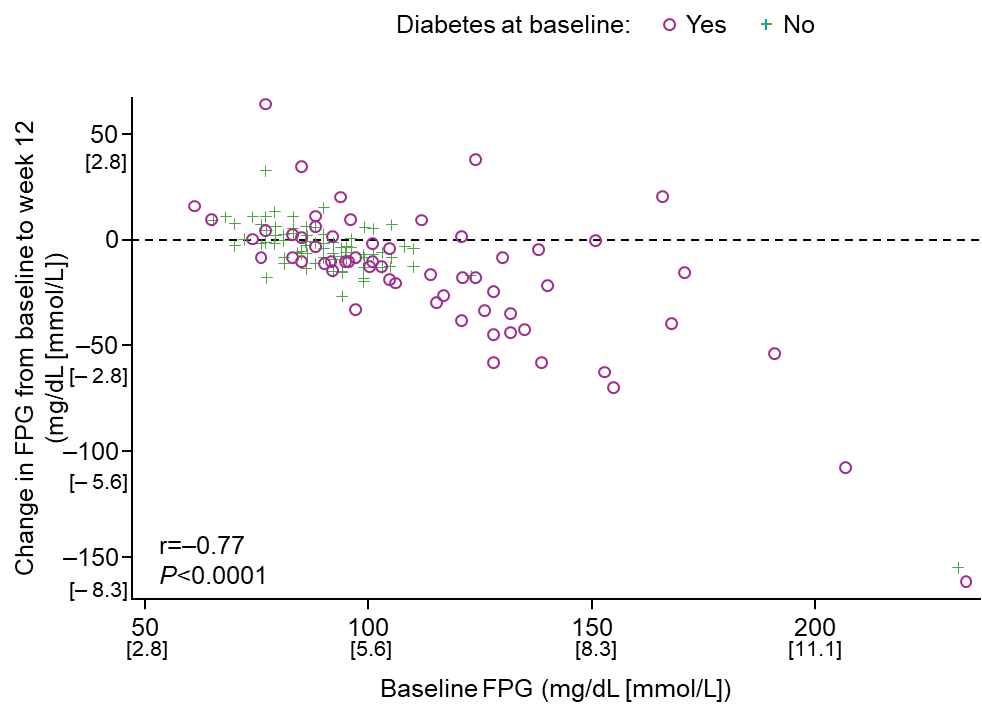


b)


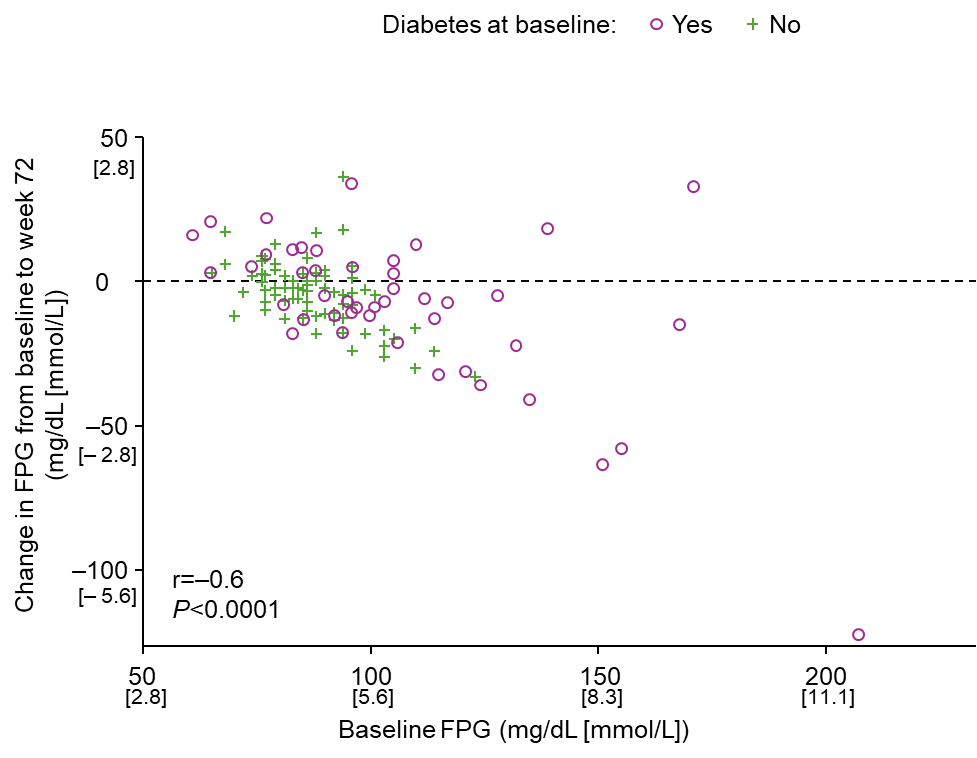


c)


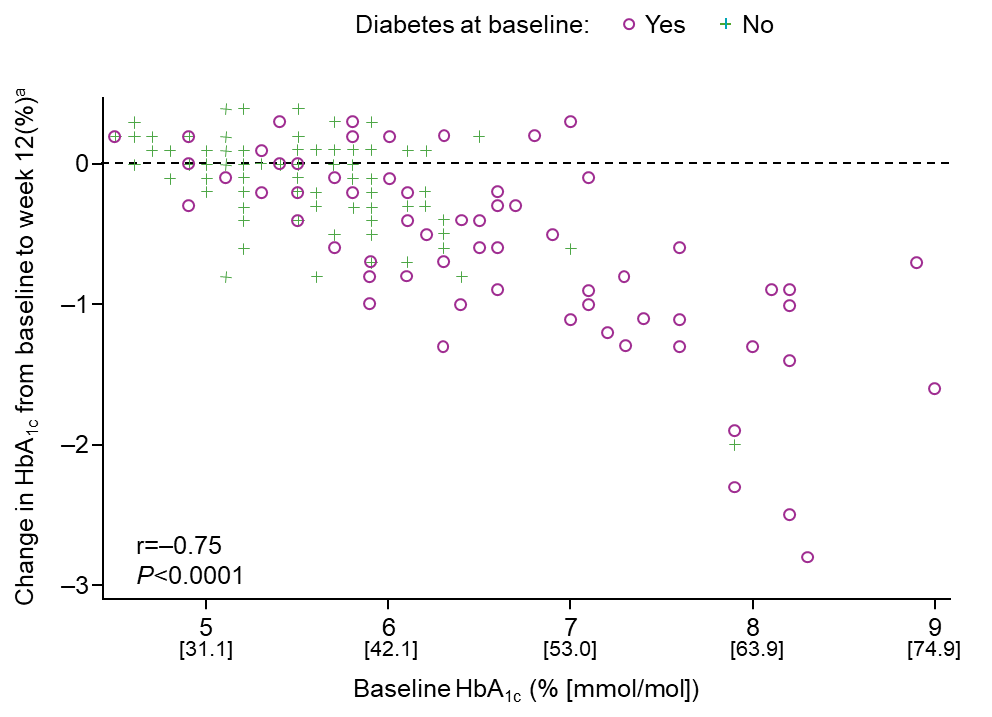


d)


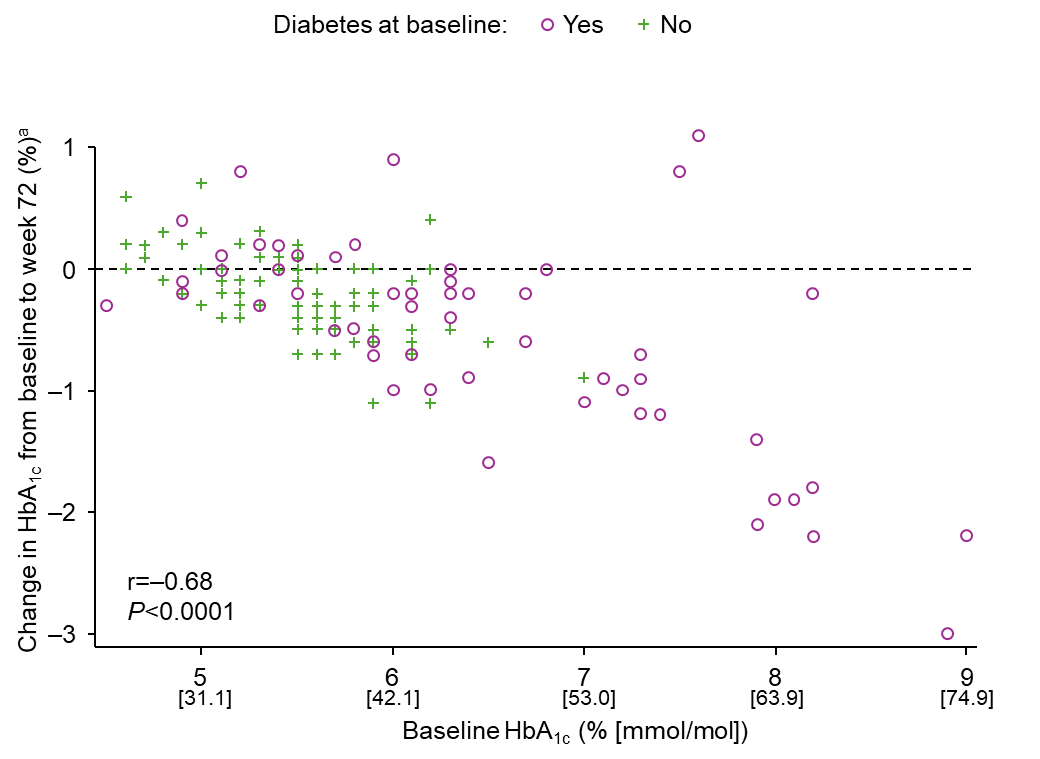


^a^SI units not calculable

Supplementary Figure 12. Mean a) FPG and b) HbA_1c_ over time, according to changes in antihyperglycemic medication use during the studies

a)


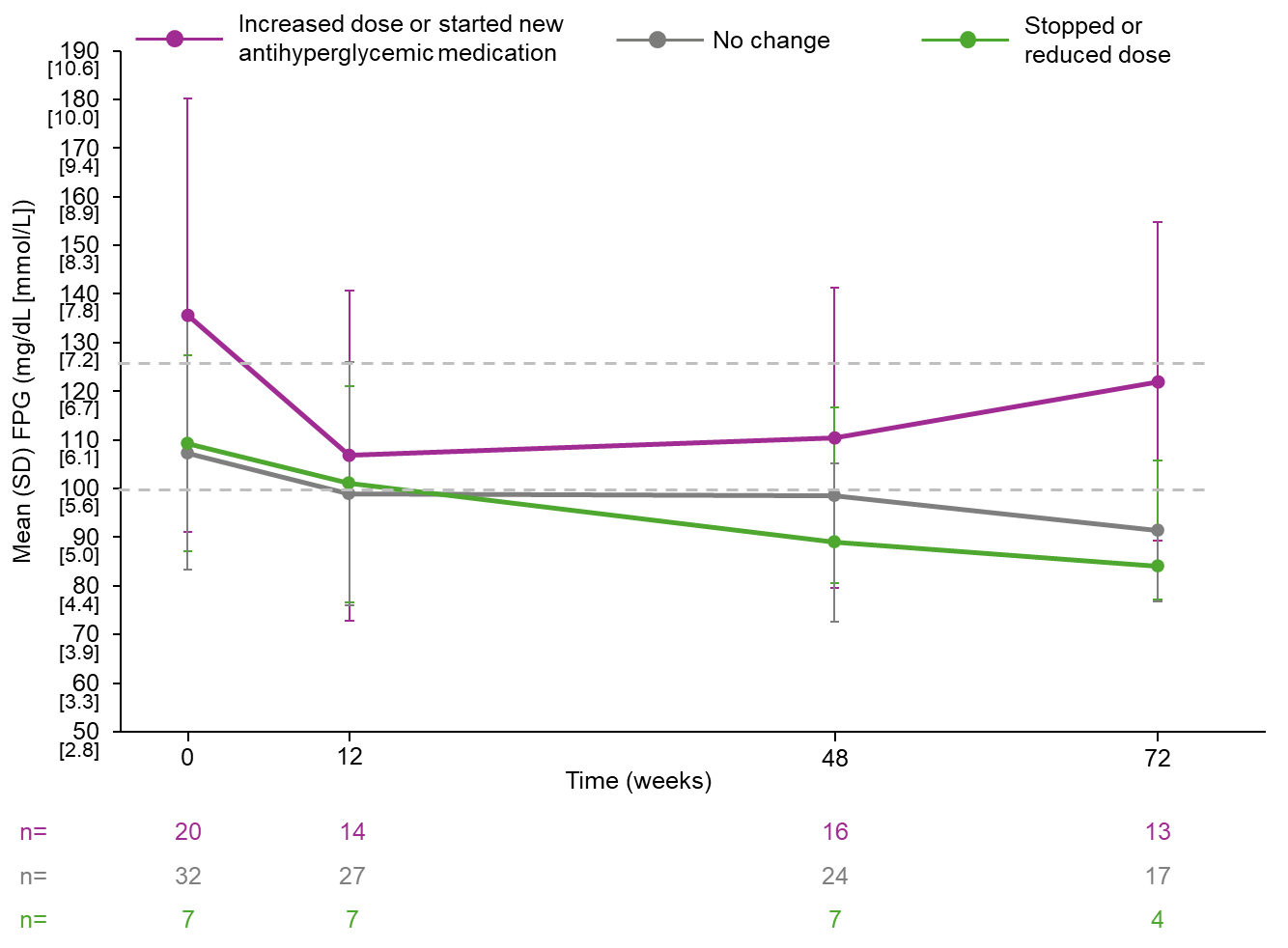


b)


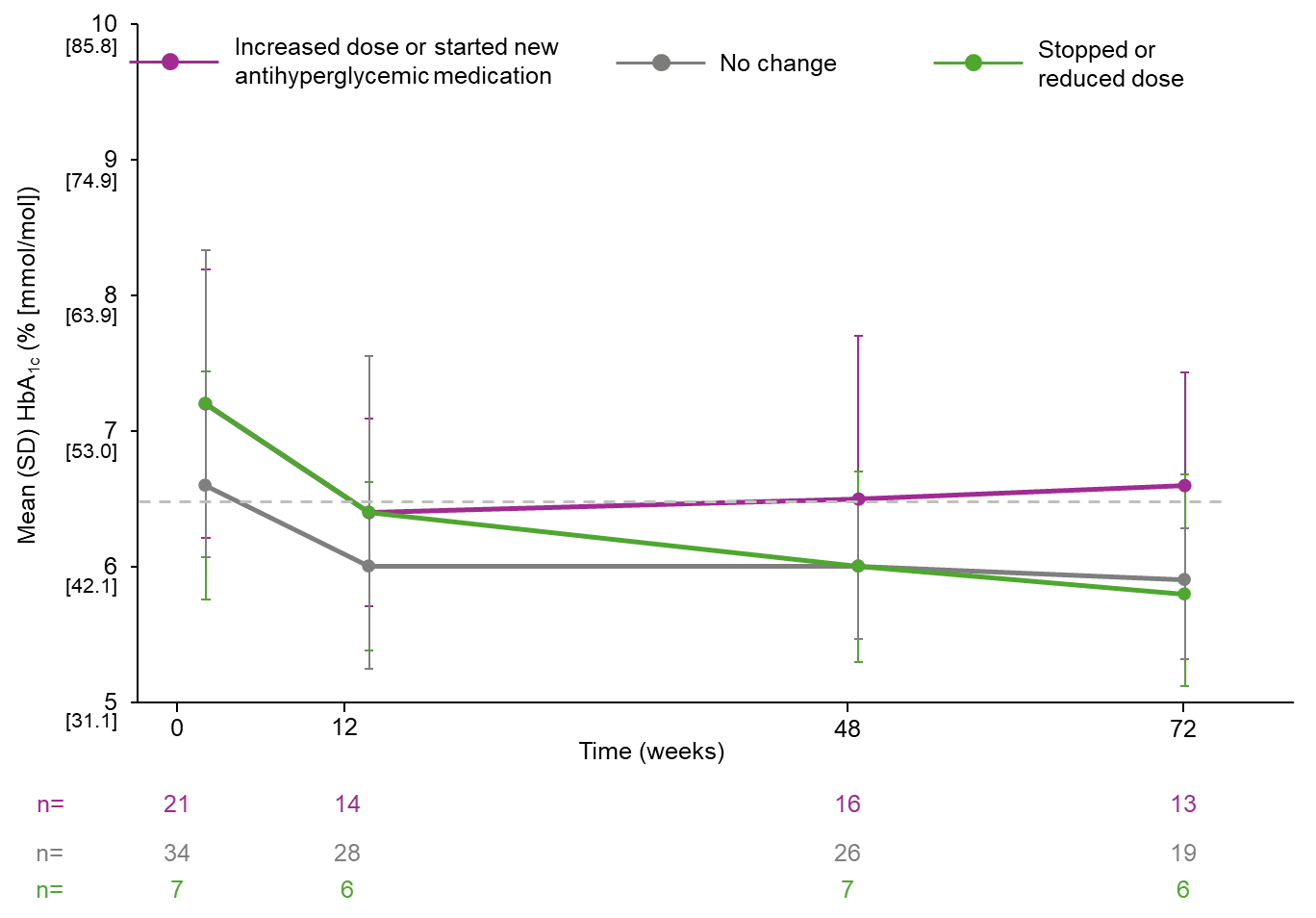


For part a, the dashed gray lines indicate the FPG thresholds for pre-diabetes (100 mg/dL [5.6 mmol/L]) and diabetes (126 mg/dL [7.0 mmol/L]). For part b, the dashed gray line indicates the HbA_1c_ threshold for diabetes (6.5% [47.5 mmol/mol])

Supplementary Figure 13. Mean FPG levels at a) W12, b) W48, and c) W72, and mean HbA_1c_ levels at d) W12, e) W48, and f) W72, over time in patients with diabetes at baseline, by mUFC control


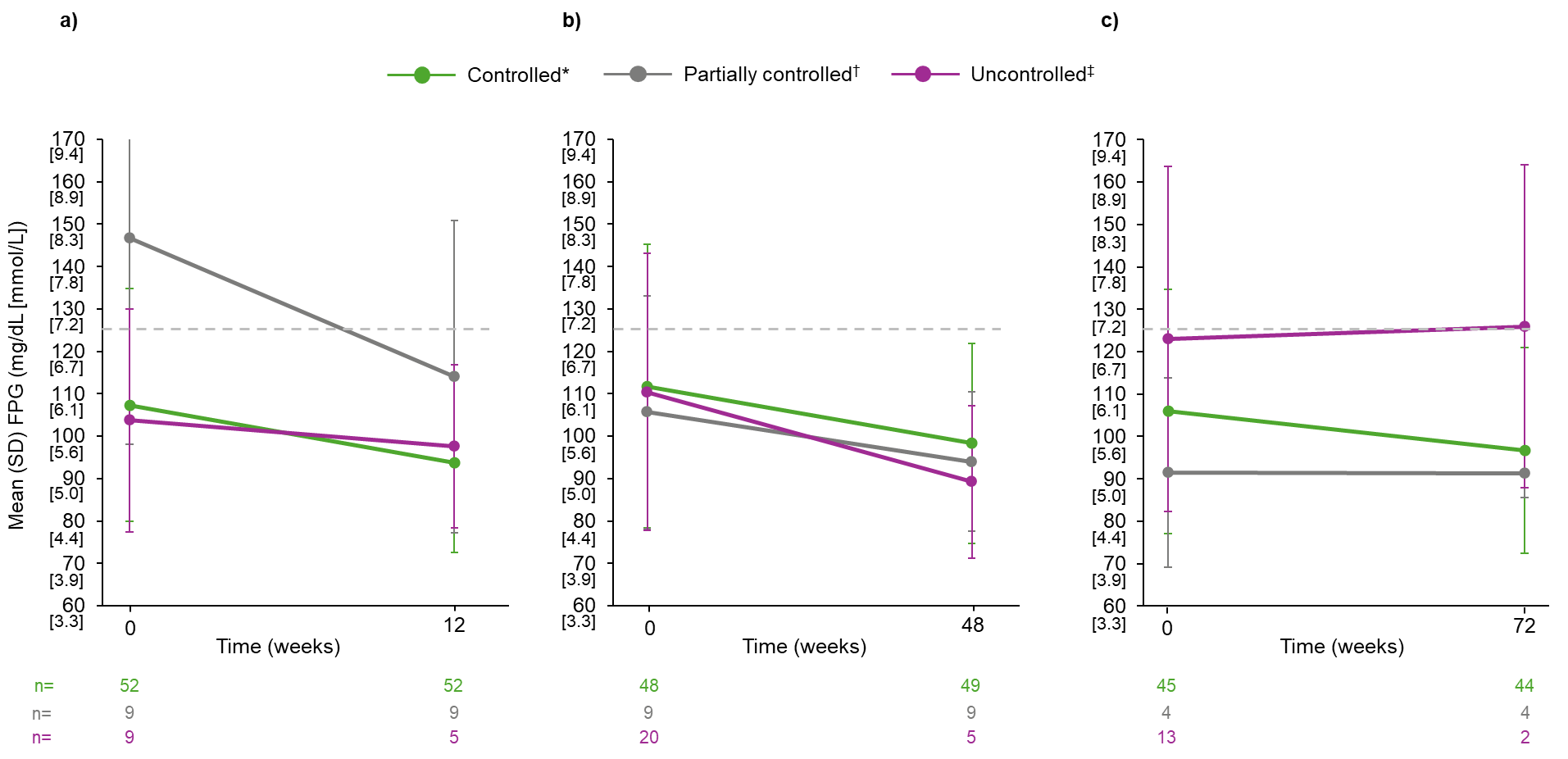


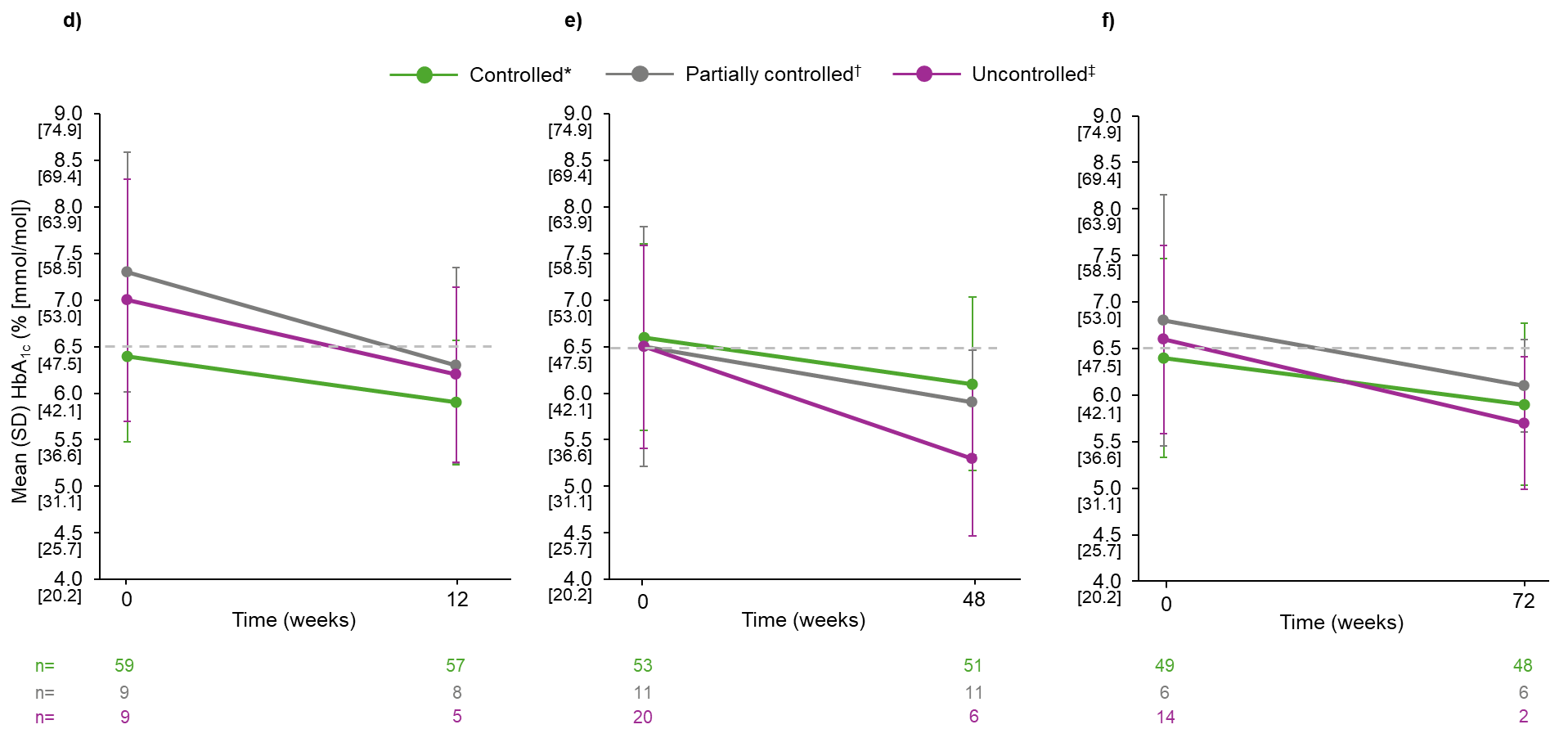


*mUFC ≤ULN; ^†^mUFC >ULN but ≥50% decrease from baseline; ^‡^mUFC>ULN and <50% decrease from baseline. For parts a–c, the dashed gray lines indicate the FPG thresholds for pre-diabetes (100 mg/dL [5.6 mmol/L]) and diabetes (126 mg/dL [7.0 mmol/L]). For parts d–f, the dashed gray lines indicate the HbA_1c_ threshold for diabetes (6.5% [47.5 mmol/mol])

Supplementary Figure 14. Changes in antihyperglycemic medication use according to mUFC control in a) patients with diabetes at baseline and taking antihyperglycemic medication and b) patients without diabetes who started antihyperglycemic medication during the studies

a)


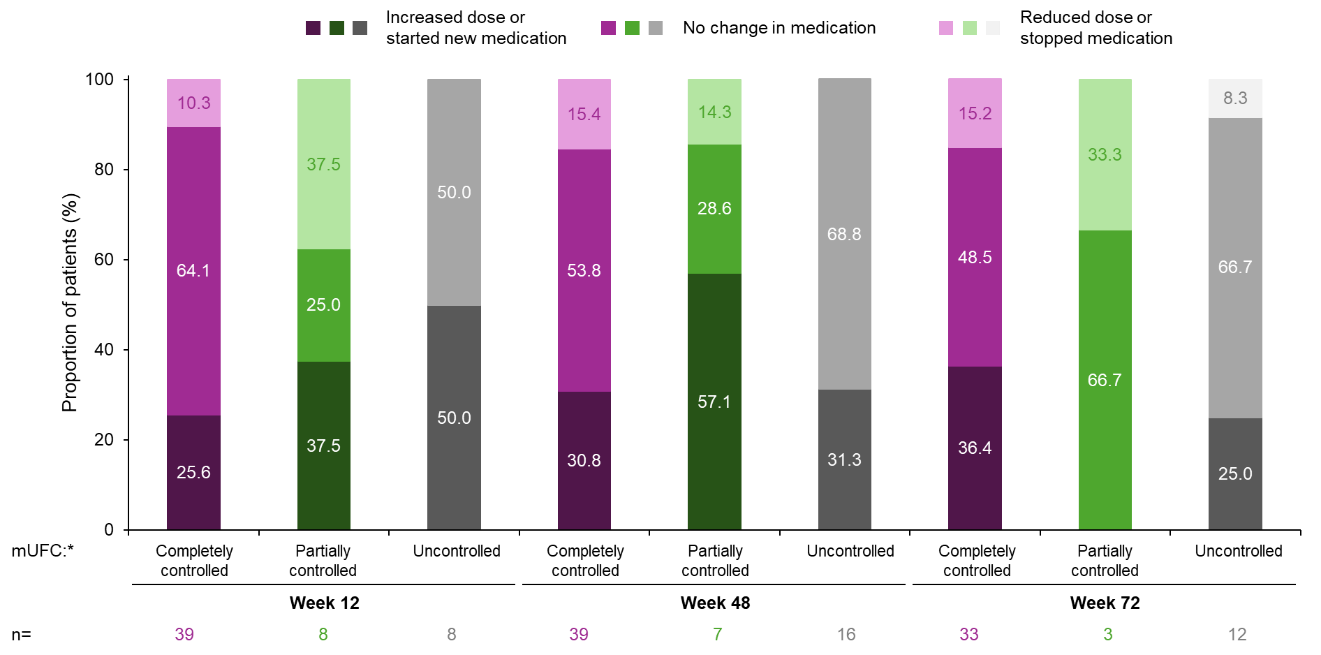


b)


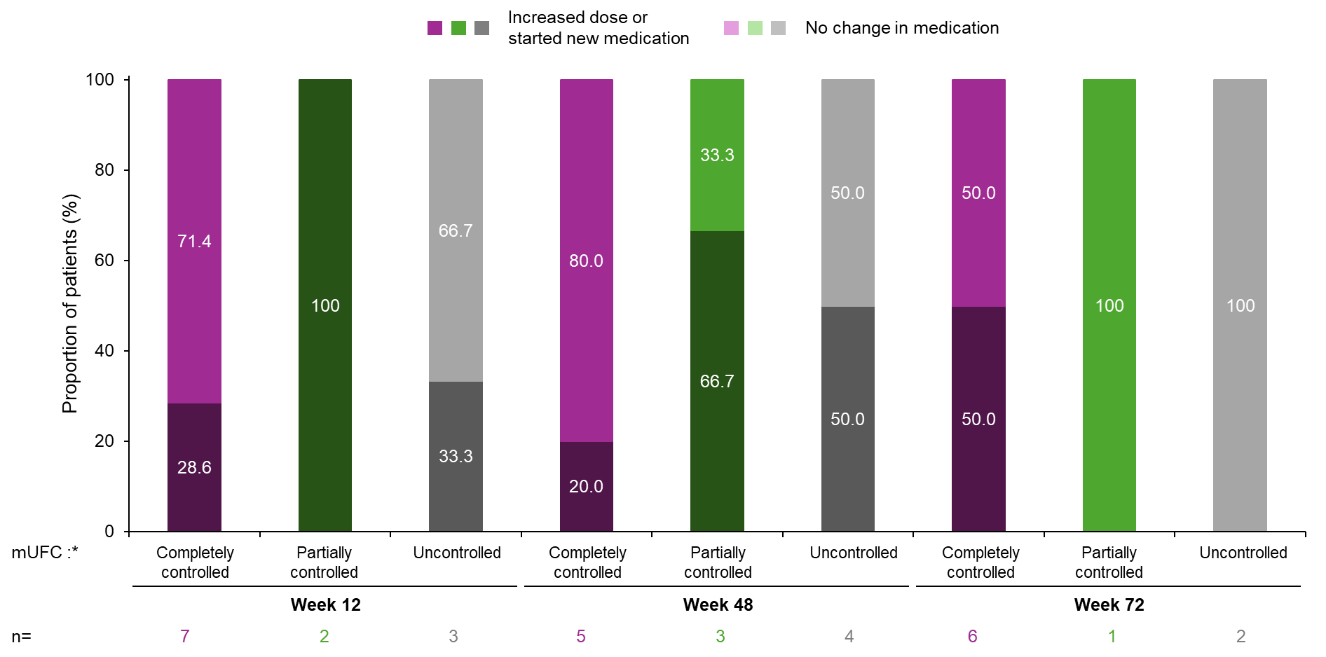


*Controlled: mUFC ≤ULN; partially controlled: mUFC >ULN but ≥50% decrease from baseline; uncontrolled: mUFC>ULN and <50% decrease from baseline

Reductions in mean (SD) weight, waist circumference, and BMI were observed from baseline to W12 in patients with and without diabetes at baseline (respectively, –1.2 [3.3] and –0.6 [3.3] kg, –1.7 [5.5] and –0.8 [6.6] cm, –0.5 [1.8] and –0.2 [1.2] kg/m^2^); reductions were maintained over long-term treatment in both subgroups (Supplementary Figure 15). There was a weak correlation between change in HbA_1c_ and change in weight from baseline to W72 in patients without diabetes at baseline (r=0.25, *P*=0.02), but not in those with diabetes (r=0.17, *P*=0.202). There was no correlation between change in FPG and change in weight from baseline to W72 in patients with and without diabetes at baseline (r=0.24, *P*=0.097 and r=0.02, *P*=0.89, respectively).

Supplementary Figure 15. Mean a) weight, b) waist circumference, and c) BMI over time, by presence/absence of diabetes at baseline


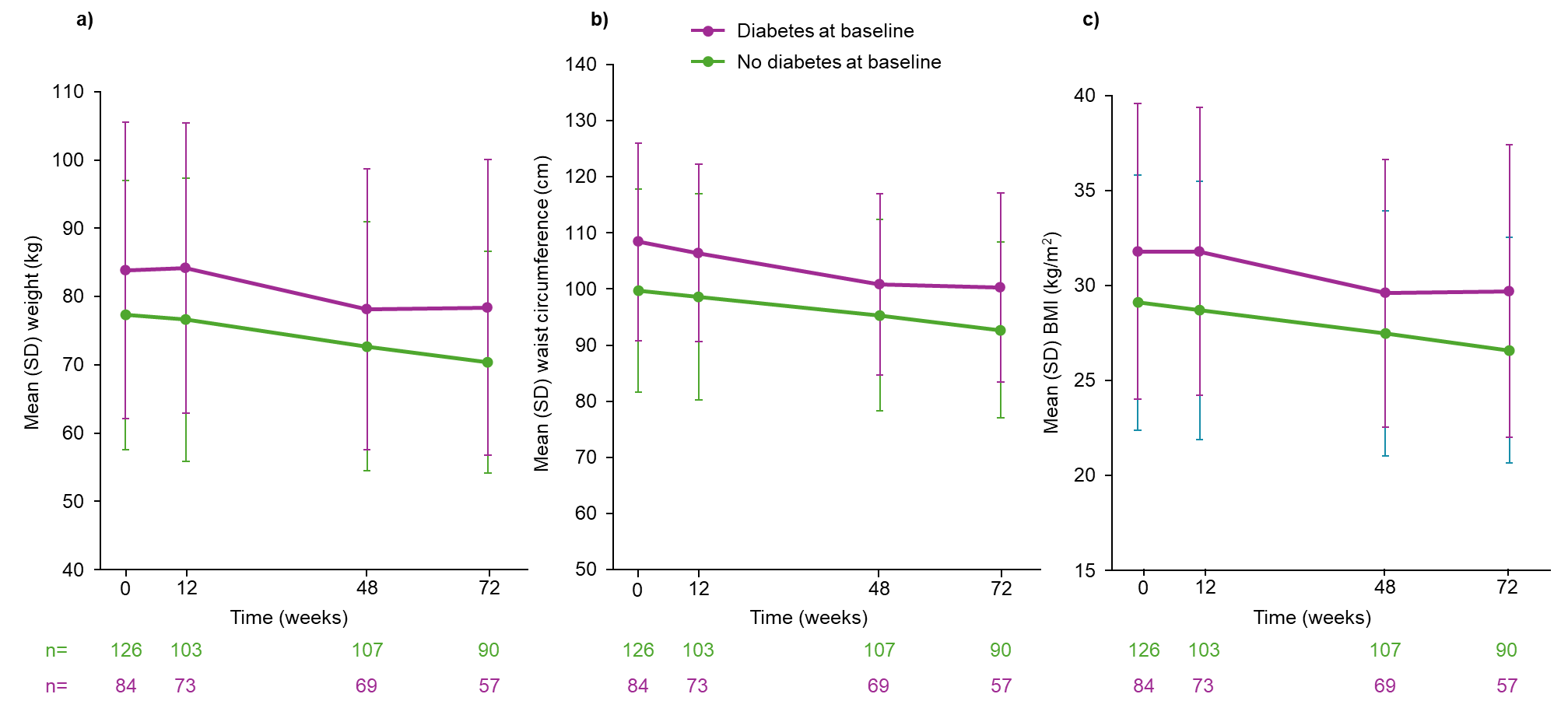

Supplement: Supplementary file 1 — Supplementary Material 1 [file 11102_2024_1471_MOESM1_ESM.docx]
